# Supplementary material for: Dengue pre-vaccination screening test evaluation for the use of dengue vaccine in an endemic area
Source: PLoS One. 2021 Sep 10;16(9):e0257182. doi: 10.1371/journal.pone.0257182 (PMC8432984; doi:10.1371/journal.pone.0257182)
Supplement: S1 Table — (DOCX) [file pone.0257182.s003.docx]

**S1 Table. Seroprevalence of DENV by PRNT (NT50, NT80 and NT90), IgG ELISA, and IgG rapid test.**

| **Characteristics** | **Seropositive by NT50** | **Seropositive by NT80** | **Seropositive by NT90** | **Seropositive by IgG ELISA** | **Seropositive  by IgG rapid test** |
| --- | --- | --- | --- | --- | --- |
|  |  |  |  |  |  |
| **Overall** | 94 (81.7%) | 86 (74.8%) | 79 (68.7%) | 82 (71.3%) | 33 (28.7%) |
| **Gender** |  |  |  |  |  |
| Male | 58 (86.6%) | 55 (82.1%) | 50 (74.6%) | 50 (74.6%) | 20 (29.9%) |
| Female | 36 (75.0%) | 31 (64.6%) | 29 (60.4%) | 32 (66.7%) | 13 (27.1%) |
| **Age** |  |  |  |  |  |
| Oct-14 | 26 (68.4%) | 22 (57.9%) | 22 (57.9%) | 21 (55.3%) | 10 (26.3%) |
| 15-18 | 32 (80.0%) | 29 (72.5%) | 26 (65.0%) | 27 (67.5%) | 8 (20.0%) |
| 19-22 | 36 (97.3%) | 35 (94.6%) | 31 (83.8%) | 34 (91.9%) | 15 (40.5%) |
