# Supplementary material for: Dengue pre-vaccination screening test evaluation for the use of dengue vaccine in an endemic area
Source: PLoS One. 2021 Sep 10;16(9):e0257182. doi: 10.1371/journal.pone.0257182 (PMC8432984; doi:10.1371/journal.pone.0257182)
Supplement: Attachment — Submitted filename: PONE-D-21-09490.pdf [file pone.0257182.s005.pdf]

PLOS ONE

Dengue pre-vaccination screening test evaluation for the use of dengue vaccine in an endemic area

--Manuscript Draft--

Manuscript Number:	PONE-D-21-09490
Article Type:	Research Article
Full Title:	Dengue pre-vaccination screening test evaluation for the use of dengue vaccine in an endemic area
Short Title:	Dengue pre-vaccination screening test evaluation
Corresponding Author:	Nattachai Srisawat<br>King Chulalongkorn Memorial Hospital<br>bangkok, THAILAND
Keywords:	CYD-TDV; Dengvaxia; Dengue serostatus; Prior dengue infection; Prevaccination screening test; rapid diagnostic test; ELISA; Sensitivity; Specificity
Abstract:	Background: The dengue vaccine (Dengvaxia) is only recommended in individuals who have had prior dengue infection (PDI). In this study, we aimed to perform a serosurvey to support decision-making for vaccine introduction and identify which populations to target. We also evaluated the performance of the serological tests using plaque reduction neutralization test (PRNT) as a reference test in identifying PDI to determine their suitability for pre-vaccination screening.<br>Methods: We enrolled 115 healthy individuals between 10 and 22 years of age living in Ratchaburi province, Thailand. The serum samples were tested by PRNT to measure the prevalence and concentration of serotype-specific neutralizing antibodies. The performance of the IgG rapid diagnostic test (RDT, SD Bioline, Korea) and IgG enzyme-linked immunosorbent assay (ELISA, EUROIMMUN, Germany) in identifying PDI were evaluated by using PRNT as a reference method.<br>Results: Overall, 94 (81.7%) of individuals neutralized one or more dengue serotypes at a titer threshold of equal or more than 10. Multitypic profiles were observed in 70.4% of the samples, which increased to 91.9% in subjects aged 19-22. Amongst monotypic samples, the highest proportion was reactive against DENV-1, followed by DENV-2, DENV-3, and DENV-4. The highest anti-dengue antibody titers were recorded against DENV-1 and increased with age to a geometric mean of 188.6 in the oldest age group. Both RDT and ELISA exhibited 100% specificity. The RDT demonstrated low sensitivity (35%), while the ELISA displayed greater sensitivity (87%).<br>Conclusions: Almost eighty percent of Thai adolescents and youth had already been exposed to the dengue virus. All four dengue serotypes have been widely circulating in Thailand. The dengue IgG RDTs have shown low sensitivity and might not suitable for dengue pre-vaccination screening. Based on these results, IgG ELISA could safely guide vaccination in Thailand.
Order of Authors:	Umaporn Limothai
	Sasipha Tachaboon
	Janejira Dinhuzen
	Taweewun Hunsawong
	Prapapun Ong-ajchaowlerd
	Butsaya Thaisomboonsuk
	Supachoke Trongkamolchai
	Mananya Wanpaisitkul
	Chatchai Chulapornsiri
	Anongrat Tiawilai
	Thawat Tiawilai

Powered by Editorial Manager® and ProduXion Manager® from Aries Systems Corporation

	Terapong Tantawichien
	Usa Thisyakorn
	Nattachai Srisawat
**Additional Information:**	
**Question**	**Response**
**Financial Disclosure**<br><br>Enter a financial disclosure statement that describes the sources of funding for the work included in this submission. Review the submission guidelines for detailed requirements. View published research articles from *PLOS ONE* for specific examples.<br><br>This statement is required for submission and **will appear in the published article** if the submission is accepted. Please make sure it is accurate.<br><br>**Unfunded studies**<br>Enter: *The author(s) received no specific funding for this work.*<br><br>**Funded studies**<br>Enter a statement with the following details:<br>• Initials of the authors who received each award<br>• Grant numbers awarded to each author<br>• The full name of each funder<br>• URL of each funder website<br>• Did the sponsors or funders play any role in the study design, data collection and analysis, decision to publish, or preparation of the manuscript?<br>• **NO** - Include this sentence at the end of your statement: *The funders had no role in study design, data collection and analysis, decision to publish, or preparation of the manuscript.*<br>• **YES** - Specify the role(s) played.<br><br>* typeset	Umaporn Limothhai received funding from the Second Century Fund (C2F), Chulalongkorn University.<br>Nattachai Srisawat received funding from the Jongkolneenithi foundation, the Medical Association of Thailand, the Tropical Medicine Cluster, Chulalongkorn University and Ratchadaphiseksomphot Endowment Fund, Chulalongkorn University.<br>The funders had no role in study design, data collection and analysis, decision to publish, or preparation of the manuscript.
**Competing Interests**<br><br>Use the instructions below to enter a competing interest statement for this submission. On behalf of all authors, disclose any competing interests that could be perceived to bias this	The authors have declared that no competing interests exist.

work—acknowledging all financial support and any other relevant financial or non-financial competing interests.<br><br>This statement is required for submission and **will appear in the published article** if the submission is accepted. Please make sure it is accurate and that any funding sources listed in your Funding Information later in the submission form are also declared in your Financial Disclosure statement.<br><br>View published research articles from *PLOS ONE* for specific examples.<br><br>**NO authors have competing interests**<br><br>Enter: *The authors have declared that no competing interests exist.*<br><br>**Authors with competing interests**<br><br>Enter competing interest details beginning with this statement:<br><br>*I have read the journal's policy and the authors of this manuscript have the following competing interests: [insert competing interests here]*<br><br>* typeset	
**Ethics Statement**<br><br>Enter an ethics statement for this submission. This statement is required if the study involved:<br><br>• Human participants<br>• Human specimens or tissue<br>• Vertebrate animals or cephalopods<br>• Vertebrate embryos or tissues<br>• Field research<br><br>Write "N/A" if the submission does not require an ethics statement.	The study was conducted according to the Helsinki Declaration and Good Clinical Practice guidelines. The study protocol was approved by the ethical committee of Banpong hospital (REC 008/2562) and Potharam hospital (REC 31/2562). The written informed consent was obtained from subjects ≥18 years old and parents of subjects <18 years old.

General guidance is provided below. Consult the submission guidelines for detailed instructions. **Make sure that all information entered here is included in the Methods section of the manuscript.**<br><br>**Format for specific study types**<br><br>**Human Subject Research (involving human participants and/or tissue)**<br>• Give the name of the institutional review board or ethics committee that approved the study<br>• Include the approval number and/or a statement indicating approval of this research<br>• Indicate the form of consent obtained (written/oral) or the reason that consent was not obtained (e.g. the data were analyzed anonymously)<br><br>**Animal Research (involving vertebrate animals, embryos or tissues)**<br>• Provide the name of the Institutional Animal Care and Use Committee (IACUC) or other relevant ethics board that reviewed the study protocol, and indicate whether they approved this research or granted a formal waiver of ethical approval<br>• Include an approval number if one was obtained<br>• If the study involved *non-human primates*, add *additional details* about animal welfare and steps taken to ameliorate suffering<br>• If anesthesia, euthanasia, or any kind of animal sacrifice is part of the study, include briefly which substances and/or methods were applied<br><br>**Field Research**<br><br>Include the following details if this study involves the collection of plant, animal, or other materials from a natural setting:<br>• Field permit number<br>• Name of the institution or relevant body that granted permission	
**Data Availability**<br><br>Authors are required to make all data underlying the findings described fully	Yes - all data are fully available without restriction

available, without restriction, and from the time of publication. PLOS allows rare exceptions to address legal and ethical concerns. See the PLOS Data Policy and FAQ for detailed information.<br><br>A Data Availability Statement describing where the data can be found is required at submission. Your answers to this question constitute the Data Availability Statement and **will be published in the article**, if accepted.<br><br>**Important:** Stating 'data available on request from the author' is not sufficient. If your data are only available upon request, select 'No' for the first question and explain your exceptional situation in the text box.<br><br>Do the authors confirm that all data underlying the findings described in their manuscript are fully available without restriction?	
**Describe where the data may be found in full sentences. If you are copying our sample text, replace any instances of XXX with the appropriate details.**<br><br>• If the data are **held or will be held in a public repository**, include URLs, accession numbers or DOIs. If this information will only be available after acceptance, indicate this by ticking the box below. For example: *All XXX files are available from the XXX database (accession number(s) XXX, XXX.*).<br>• If the data are all contained **within the manuscript and/or Supporting Information files**, enter the following: *All relevant data are within the manuscript and its Supporting Information files.*<br>• If neither of these applies but you are able to provide **details of access elsewhere**, with or without limitations, please do so. For example:<br><br>*Data cannot be shared publicly because of [XXX]. Data are available from the XXX Institutional Data Access / Ethics*	All relevant data are within the manuscript and its Supporting Information files.

Committee (contact via XXX) for researchers who meet the criteria for access to confidential data.<br><br>*The data underlying the results presented in the study are available from (include the name of the third party and contact information or URL).*<br>• This text is appropriate if the data are owned by a third party and authors do not have permission to share the data.<br><br>* typeset	
Additional data availability information:	

Powered by Editorial Manager® and ProduXion Manager® from Aries Systems Corporation

Manuscript Click here to access/download;Manuscript;MS1st-edit ref.docx

Dengue pre-vaccination screening test evaluation for the use of dengue vaccine in an endemic area

Umaporn Limothai[1,2], Sasipha Tachaboon[1,2,] Janejira Dinhuzen[1,2], Taweewun Hunsawong[3], Prapapun Ong-ajchaowlerd[3], Butsaya Thaisomboonsuk[3], Supachoke Trongkamolchai[4], Mananya Wanpaisitkul[4], Chatchai Chulapornsiri[4], Anongrat Tiawilai[5], Thawat Tiawilai[5], Terapong Tantawichien[6,7], Usa Thisyakorn[7], Nattachai Srisawat[1,2,7-10*]

1. Excellence Center for Critical Care Nephrology, King Chulalongkorn Memorial Hospital, Bangkok, Thailand.
2. Critical Care Nephrology Research Unit, Chulalongkorn University, Bangkok, Thailand.
3. Department of Virology, Armed Forces Research Institute of Medical Sciences, Bangkok, Thailand.
4. Banpong Hospital, Ratchaburi, Thailand.
5. Photharam Hospital, Ratchaburi, Thailand.
6. Division of Infectious Diseases, Department of Medicine, Faculty of Medicine, Chulalongkorn University, Bangkok, Thailand.
7. Tropical Medicine Cluster, Chulalongkorn University, Bangkok, Thailand.
8. Division of Nephrology, Department of Medicine, Faculty of Medicine, King Chulalongkorn Memorial Hospital, Bangkok 10330, Thailand.
9. Center for Critical Care Nephrology, The CRISMA Center, Department of Critical Care Medicine, University of Pittsburgh, School of Medicine, Pittsburgh, PA, USA.
10. Academy of Science, Royal Society of Thailand, Bangkok, Thailand.

Keyword: CYD-TDV; Dengvaxia; Dengue serostatus; Prior dengue infection Prevaccination screening test; rapid diagnostic test; ELISA; Sensitivity; Specificity

Short title: Dengue pre-vaccination screening test evaluation

***Corresponding Author:** Assoc. Prof. Dr. Nattachai Srisawat
Excellence Center for Critical Care Nephrology, Thai Red Cross Society, King Chulalongkorn Memorial Hospital
1873, Rama 4 Rd., Lumphini, Pathumwan, Bangkok 10330
Phone (662) – 2564000 Ext.3597 Email: drnattachai@yahoo.com

Abstract

Background: The dengue vaccine (Dengvaxia) is only recommended in individuals who have had prior dengue infection (PDI). In this study, we aimed to perform a serosurvey to support decision-making for vaccine introduction and identify which populations to target. We also evaluated the performance of the serological tests using plaque reduction neutralization test (PRNT) as a reference test in identifying PDI to determine their suitability for pre-vaccination screening.

Methods: We enrolled 115 healthy individuals between 10 and 22 years of age living in Ratchaburi province, Thailand. The serum samples were tested by PRNT to measure the prevalence and concentration of serotype-specific neutralizing antibodies. The performance of the IgG rapid diagnostic test (RDT, SD Bioline, Korea) and IgG enzyme-linked immunosorbent assay (ELISA, EUROIMMUN, Germany) in identifying PDI were evaluated by using PRNT as a reference method.

Results: Overall, 94 (81.7%) of individuals neutralized one or more dengue serotypes at a titer threshold of equal or more than 10. Multitypic profiles were observed in 70.4% of the samples, which increased to 91.9% in subjects aged 19-22. Amongst monotypic samples, the highest proportion was reactive against DENV-1, followed by DENV-2, DENV-3, and DENV-4. The highest anti-dengue antibody titers were recorded against DENV-1 and increased with age to a geometric mean of 188.6 in the oldest age group. Both RDT and ELISA exhibited 100% specificity. The RDT demonstrated low sensitivity (35%), while the ELISA displayed greater sensitivity (87%).

Conclusions: Almost eighty percent of Thai adolescents and youth had already been exposed to the dengue virus. All four dengue serotypes have been widely circulating in Thailand. The dengue IgG RDTs have shown low sensitivity and might not suitable for dengue pre-

vaccination screening. Based on these results, IgG ELISA could safely guide vaccination in Thailand.

Introduction

Dengue is a mosquito-borne viral disease that has rapidly spread across the tropical and subtropical regions. Dengue virus consists of four serotypes with antigenic differences (DENV-1 to 4) [1]. It has a broad clinical spectrum that includes both severe and non-severe clinical manifestations [2]. The first dengue vaccine, a live, attenuated, tetravalent dengue vaccine (Dengvaxia or CYD-TDV) developed by Sanofi Pasteur, was licensed and available in some countries. The efficacy trials showed that Dengvaxia protected against severe dengue in people who had exposure to dengue prior to vaccination. However, there was evidence of a higher longer-term risk of severe dengue in vaccinated persons who had not been exposed to dengue [3]. World Health Organization (WHO) Strategic Advisory Group of Experts (SAGE) recommends using Dengvaxia in children 9-16 years old living in highly dengue-endemic areas ~~[4]~~.. Furthermore, in defining people to be targeted for vaccination, prior dengue infection (PDI), as measured by seroprevalence, should be approximately 70% or more [4]. To minimize potentially harmful effects, we should ensure that the age group targeted for vaccination has enough pre-existing dengue immunity to benefit from the vaccine (add reference).

Age-stratified serosurveys are currently the best method for selecting populations appropriate for vaccination. Immunoglobulin G (IgG)-class antibodies to dengue are useful serological markers for PDI as they can persist over an individual's lifetime [5]. Plaque reduction neutralization test (PRNT) is the most specific serological test for determining type-specific neutralizing antibodies to an infecting virus. This technique can be used to confirm the presence of the neutralization antibodies against Flaviviruses in the serum samples. ~~This technique can be used to confirm the infecting flavivirus from a convalescent serum sample~~ [6, 7]. However, PRNT is a labor-intensive, relatively costly test, and it might not be convenient for routine surveillance

Formatted: Font color: Red

Formatted: Font color: Red

Formatted: Strikethrough

purposes. ~~An~~ Commercial IgG rapid diagnostic test (RDT) and ~~an~~ IgG enzyme-linked immunosorbent assay (ELISA) are commonly used to diagnose acute dengue infection [8]. Nonetheless, the performance data for these tests for the identification of PDI are still limited (reference). Explain more/provide some data from previous studies.

In this study, we aimed to perform a comparative serosurvey to support decision-making for dengue vaccine introduction in ……. ages. We also evaluated the performance of the serological tests as a pre-screening test in identifying PDI to determine their suitability for pre-vaccination screening in Thailand.

Materials and methods

Ethics Statement

The study was conducted according to the Helsinki Declaration and Good Clinical Practice guidelines. The study protocol was approved by the ethical committee of Banpong hospital (REC 008/2562) and Potharam hospital (REC 31/2562). The written informed consent was obtained from subjects ≥18 years old and parents of subjects <18 years old.

Cells and Viruses

In this section, you must add all cells and viruses that you have used in this experiment including the passage numbers, source of the cells and viruses.

Study Location and Population

The study was conducted in Banpong and Potharam district, Ratchaburi province, an urban center located in the western part of Thailand. Healthy children (you must include the criteria here, how do you considered them healthy?) between 9 and 22 years of age at the enrollment and residing in Banpong or Potharam district were eligible to participate. Using the sample size calculation by Malhotra RK. and Indrayan A. [9], assuming 91% seroprevalence [10, 11], expected sensitivity and specificity of 95% and 95%, respectively, and desired precision of 0.05, we estimate that at least 80 samples would need to be tested. With indeterminate and invalid results, we tested 115 samples. Specimens were

Formatted: Strikethrough

Formatted: Space Before: 0.1 pt

Formatted: List Paragraph, Numbered + Level: 1 + Numbering Style: 1, 2, 3, … + Start at: 63 + Alignment: Left + Aligned at: 0.19" + Indent at: 0.61", Tab stops: 0.61", Left + 0.61", Left

Formatted: Space Before: 0.1 pt

Formatted: List Paragraph, Numbered + Level: 1 + Numbering Style: 1, 2, 3, … + Start at: 63 + Alignment: Left + Aligned at: 0.19" + Indent at: 0.61", Tab stops: 0.61", Left + 0.61", Left

Formatted: Font color: Red

Formatted: Font color: Red

Formatted: Font color: Red

Formatted: Font: 12 pt

Formatted: Left, Indent: Left: 0.11", Hanging: 0.5", No bullets or numbering, Tab stops: Not at 0.61" + 0.61"

Formatted: Font: Bold, Font color: Red

Formatted: Font color: Auto

Formatted: Font: 12 pt, Bold

Formatted: Left, Indent: Left: 0.11", Hanging: 0.5", No bullets or numbering, Tab stops: Not at 0.61" + 0.61"

Formatted: Font: Not Bold, Font color: Red

Formatted: Font: Not Bold, Font color: Red

Formatted: Font color: Red

Formatted: Font color: Red

Formatted: Font color: Red

collected from August 2019-August 2020. The blood samples were collected in anticoagulant-free vacutainer tubes. The sera were separated and maintained refrigerated at 2-8˚C. The

specimens were then transported on the day of collection to the central laboratory at Chulalongkorn hospital and stored at -80°C before testing.

Plaque Reduction Neutralization Test (PRNT)

Please add the name of companies that you purchased all the materials from them.

PRNT was performed to determine the levels of virus type-specific neutralizing (NT) antibodies using standard methods, as previously described [6, 12]. (Did you heat inactivated the serums before the experiment?) Briefly, serum was diluted in a four-fold serial dilution starting from 1:10 to 1:2,560 and separately mixed with an equal volume of each DENV serotype (Which viruses, name, culture history, passage number, which moi. Are these viruses circulating in your country?????) before incubation in a 35°C water bath for 1 hour. The mixture was then inoculated into Rhesus monkey kidney epithelial cells (LLC-MK2 cells) in duplicate wells of a 12-well plate and incubated at room temperature for 1 hour on a rocker platform. After removing of excess volume of inoculum, washed by???, the first overlay medium (which media and how much?) containing 5% heat-inactivated fetal bovine serum (company name?) in 0.9% low melting point agarose gel (LMP, Ultra PureTM LMP agarose, Invitrogen, USA) was added and incubated for 4-6 days in a 5% CO_2 incubator at 35°C before staining with 4% neutral red (Sigma, USA) in a second overlay medium (how much) with LMP and incubated for ????. The number of plaques was determined by manual counting. PRNT data were expressed as the reciprocal of the serum dilution causing 50% plaque reduction (NT50) as extrapolated by probit regression which was identified as the highest dilution that can reduce the number of plaques by 50% as compared to the number of plaques in the virus control well. Antibody titers ≥10 were considered positive for virus-neutralizing activity (Could you please explain it more? I think the calculation of PRNT is not correct. You can follow the instruction in this article to do the analysis again: doi: 10.4269/ajtmh.2009.08-0625. **This is my main concern in this manuscript??????????????**

An individual who has antibody titers <10 for the four serotypes was classified as naïve. An individual who has NT50 antibody titers ≥10 to only one serotype (no NT titer to other serotypes) was classified as monotypic.

Formatted: Font color: Red

Formatted: List Paragraph, Left, No bullets or numbering, Tab stops: Not at 0.61" + 0.61"

Formatted: Font color: Red

Formatted: Font color: Red

Formatted: Font color: Red

Formatted: Font color: Red

Formatted: Font color: Red

Formatted: Not Expanded by / Condensed by

Formatted: Font color: Red

Formatted: Font color: Red

Formatted: Font color: Red

Formatted: Font color: Red

Formatted: Font color: Red

Formatted: Font color: Red

Formatted: Font color: Red

Formatted: Font: Bold, Underline, Font color: Red

Formatted: Font: 12 pt

Formatted: Left, Indent: Left: 0.11", Hanging: 0.5", No bullets or numbering, Tab stops: Not at 0.61" + 0.61"

Formatted: Space Before: 0.1 pt

Formatted: List Paragraph, Left, Indent: Hanging: 0.5", Numbered + Level: 1 + Numbering Style: 1, 2, 3, … + Start at: 93 + Alignment: Left + Aligned at: 0.19" + Indent at: 0.61", Tab stops: 0.61", Left + 0.61", Left

Correspondingly, an individual who has NT50 antibody titers ≥10 for more than one serotype was classified as multitypic. You need to explain more about the positive and control in PRNT assay. You must determine a minimum number of plaques in positive control.

Formatted: Font color: Red

Dengue duo rapid test kit

Did you heat inactivate the serums?

Dengue nonstructural protein 1 (NS1) antigen and dengue IgG/IgM antibody were examined at the enrollment using SD Bioline Dengue Duo NS1 Ag & IgG/IgM (catalog number 11FK46, SD Bioline, Korea) according to the manufacturer's instructions. For the NS1 antigen, 100 μL of diluted or undiluted ???serum was added to the left sample well, incubated at RT?????????? and the results were read within 15-20 minutes. For IgG/IgM, 10 μL of serum was added to the right sample well, and four drops (90-120 μL) of assay diluent were added to the round assay diluent well. The results were read 15-20 minutes later. Each test included negative and positive????????? control and a test line. The appearance of both the control and test lines indicated a positive result. In contrast, the appearance of only the control line indicated a negative result [13].

Formatted: Font color: Red

Formatted: Font color: Red

Formatted: Font color: Red

Formatted: Font color: Red

You must describe how to determine the positivity and negativity in this experiment clearly. There are some good articles in this field. Just an example: doi: 10.4269/ajtmh.2010.10-0117

Dengue IgG ELISA

Anti-Dengue IgG antibodies were assessed using commercial enzyme-linked immunosorbent assay (catalog number EI266b-9601G, EUROIMMUN, Lubeck, Germany) according to the manufacturer's instructions. Briefly, serum samples were diluted at 1:101, and 100 μL of the mixture was then added to the 96-well microplates coated with highly purified DENV-2 particles. Please mention that this kit can detect DENV 1 to 4 and this kit used conserved region. After incubating for 60 minutes at room temperature???????, the wells were washed by????? How many????????, and 100 μL of enzyme conjugate (peroxidase-labeled anti-human IgG) was added to each well. After incubating for 30 minutes at RT?????????, the plates were washed by????? How many????????, and the conjugated complex was visualized by adding 100 μL of TMB solution to each well. After incubating for 15 minutes at RT??????, the reaction was stopped by

Formatted: Font color: Red

Formatted: Font color: Red

Formatted: Font color: Red

Formatted: Font color: Red

Formatted: Font color: Red

Formatted: Font color: Red

Formatted: Font color: Red

adding 100 μL of stop solution to each well. The colorimetric output was evaluated in a microtiter plate reader at 450 nm. Positive determinations were made against the provided IgG reference sera (cutoff calibrators). Antibody values > 20 relative units/mL were considered seropositive (Can you explain more about this number and how you reached it?).

Statistical Analysis

Descriptive statistics for seropositive rate and other characteristics were performed. Continuous variables are presented as the mean ± standard deviation (SD) in case of a normal distribution and as a median and interquartile range (IQR) in case of non-normally distributed variables. The comparison was performed by one-way ANOVA or Kruskal–Wallis test as appropriate. Categorical variables were presented as numbers with percentages and were compared using the Chi-square test or Fisher's exact test as appropriate. P-value <0.05 was considered significant. The geometric mean titer and the 95% CI for each age group and dengue serotype were calculated for all samples based on their DENV PRNT results. To calculate the GMT, samples with an antibody titer <10 were given the value of 5. All analyses were performed using IBM SPSS Statistics software version 22.0 (IBM Corp., Armonk, NY)

Disclaimer

Material has been reviewed by the Walter Reed Army Institute of Research. There is no objection to its presentation and/or publication. The opinions or assertions contained herein are the private views of the author, and are not to be construed as official, or as reflecting true views of the Department of the Army or the Department of Defense.

Results

Demographic data

Table 1 provides general characteristics of 115 participants included in the study. Their age ranged from 10 to 22 years, with a median of 16 years (IQR 13-20). More than half (67,

Formatted: Font color: Red

Formatted: Font color: Red

58.3%) were males. The participants were predominantly (53%) from Ban Pong District.

Table 1 Demographic data (any specific reason for these groups?????? Any specific reason for these geographical area?? Is there any available previous data from these regions???)

Characteristics	Total (N=115)
Gender, male, (N, %)	67 (58.3)
Age, years (median, range)	16 (10-22)
10-14 (N,%)	38 (33.0)
15-18 (N,%)	40 (34.8)
19-22 (N,%)	37 (32.2)
Area	
Ban Pong (N,%)	61 (53.0)
Potharam (N,%)	54 (47.0)

Formatted: Font color: Red

Formatted: Font: 12 pt

Formatted: Normal, Right, No bullets or numbering

Seroprevalence of DENV amongst participants

Serological evidence of previous exposure to DENV as denoted by NT50 titers ≥ 10 was identified in the serum samples of 94 (81.7%) participants. Seroprevalence did not significantly differ based on gender (p=0.113). To examine the DENV seroprevalence in different age groups, we classified the participant's age into three categories based on school-age, namely 10-14 years, 15-18 years, and 19-22 years (You must mention this in the methods). The data indicated that increasing age appeared to be associated with increased seropositivity (p=0.005). We also examined the seroprevalence by IgG ELISA and IgG rapid test. The results showed that the overall seropositive rate had decreased to 71.3% by IgG ELISA and 28.7% by IgG rapid test (Table 2).

Formatted: Font color: Red

Table 2 Seroprevalence of DENV by NT50 (titers ≥ 10), IgG ELISA, and IgG rapid test (It will be grate to add graph for this data)

Formatted: Font color: Red

Characteristics	Seropositive by NT50	Seropositive by IgG ELISA	Seropositive by IgG rapid test
**Overall**	94 (81.7%)	82 (71.3%)	33 (28.7%)
**Gender**			
Male	58 (86.6%)	50 (74.6%)	20 (29.9%)
Female	36 (75.0%)	32 (66.7%)	13 (27.1%)
**Age**			
10-14	26 (68.4%)	21 (55.3%)	10 (26.3%)
15-18	32 (80.0%)	27 (67.5%)	8 (20.0%)
19-22	36 (97.3%)	34 (91.9%)	15 (40.5%)

Data are presented as n (%)

Neutralizing antibody profile distribution stratified by age

Samples were categorized according to the NT50 profile. Multitypic profiles were observed in 70.4% of the subjects, with 55.3% in those aged 10-14 years old, 65.0% in the 15-18 years old, and 91.9% in the 19-22 years old (Fig 1).

Fig 1. Prevalence of individuals with naïve (NT50 titer<10), monotypic (NT50 titer≥10 against to only one dengue serotype), or multitypic (NT50 titer≥10 against more than one serotypes) neutralizing antibody profile, by age group

There were 31.6% naïve subjects in the 10-14 years old group, 20.0% of the 15-18 years old group and 2.7% of the 19-22 years old groups, and 18.3% of the overall sample had no detectable neutralizing dengue antibodies at the ten thresholds. (Table 2)

Among monotypic samples, the highest proportion of samples were reactive against DENV-2, followed by DENV-1, DENV-3, and DENV-4, a trend observed in the 19-22 years old age groups. The proportion of samples that were reactive against DENV-1 and DENV-2 were evenly distributed amongst the 10–14 and 15–18 years old age groups.

The distribution of geometric mean NT50 titer (GMT) among the ages 10-22 years old was shown in Fig 2A. DENV-1 had the highest overall GMT (82.2), followed by DENV-2 (74.5), DENV-3 (59.3), and DENV-4 (34.2). The trends were also observed in the 10-14 years old age group (Fig 2B, the GMT were 43.1, 42.8, 38.8, and 25.8 respectively), and in the 19-22 years old age group (Fig 2D), the GMT was 188.6, 126.0, 124.9, and 51.7 respectively). For the 15-18 years old age groups (Fig 2C), DENV-2 had the highest GMT (77.7), followed by DENV-1 (70.5), DENV-3 (44.6), and DENV-4 (30.4).

Fig 2. Dengue serotype-specific geometric mean titer (GMT) by age-group.
The long solid line indicates the threshold for seropositive (an NT50 titer ≥10).

Performance of the dengue IgG rapid test and dengue IgG ELISA for determination of dengue serostatus

We assessed the performance of the IgG rapid test and IgG ELISA for detection of dengue pre-exposure using the PRNT50 as the reference assay. Of the 94 patients classified as seropositive by PRNT (titers ≥ 10), 61 (64.9%) were tested negative by dengue IgG rapid test, and 12 (12.8%) were tested negative by dengue IgG ELISA. For the dengue IgG rapid test, sensitivity was 35.1%, and specificity was 100%. When we evaluated the dengue IgG ELISA to detect prior dengue infection, specificity was unchanged, but sensitivity increased to 87.2% compared to IgG rapid test. Positive predictive values (PPV) for both tests were equal (100%). Negative predictive values (NPV) were 25.6% and 63.6% for dengue rapid test and IgG ELISA, respectively (Table 3).

Table 3. Performance of the dengue IgG rapid test and dengue IgG ELISA for dengue serostatus determination using the plaque reduction neutralization test (PRNT) as the reference assay.

Test	Dengue rapid test (IgG)		Dengue ELISA (IgG)	
	Positive	Negative	Positive	Negative
Positive PRNT (n,%)	33 (35.1%)	61 (64.9%)	82 (87.2%)	12 (12.8%)
Negative PRNT (n,%)	0 (0.0%)	21 (100.0%)	0 (0.0)	21 (100.0%)
Sensitivity	35.1%		87.2%	
Specificity	100.0%		100.0%	
Positive predictive value	100.0%		100.0%	
Negative predictive value	25.6%		63.6%	

The relationship between NT50 titer and dengue IgG level (relative RU/mL by ELISA) and was also examined. The result showed that NT50 titer had a positive correlation with IgG level ($r=0.701$, $p<0.001$ for DENV-1, $r=0.771$, $p<0.001$ for DENV-2, $r=0.667$, $p<0.001$ for

DENV-3, and r=0.725, p<0.001 for DENV-4). The distribution of dengue IgG level and NT50 titer by age was demonstrated in Fig 3.

Fig 3. Distribution of dengue IgG level (relative units per milliliter by ELISA) and NT50 titer by age

The dashed line indicates the threshold for dengue IgG ELISA (20 RU/ml) and NT50 (a titer of 10).

Discussion

We conducted a dengue seroprevalence study, which identified serological evidence for the circulation of all four dengue serotypes among adolescents and youth in Ratchaburi province, Thailand. This study generated data on serotype-specific prevalence in areas where little data were previously available. The result indicated that approximately 82% of individuals neutralized one or more dengue serotypes by PRNT50. The proportion of individuals with exposure to more than one serotype was found to be increased with age. They were more likely to have been infected with DENV-1, followed by DENV-2, DENV-3, and DENV-4.

The seroprevalence data among Thais individuals living in central and southern Thailand (age ranged from 6 months to 60 years) indicated that the overall seropositive rate for anti-DENV IgG antibodies by ELISA (catalog numbers EI266b-9601G, EUROIMMUN, LuÈbeck, Germany) was 79%, which was similar to this study [10]. More recently, a higher seroprevalence rate was observed in northeastern Thailand (approximately 90%, age ranged from 5 to 93 years) by the same ELISA kit. Besides, the seroprevalence was 100% for participants above the age of 25 years [11]. A previous study in healthy children in Bangkok

(the capital city of Thailand) indicated that 9-23% of children aged between 9-18 months had

neutralizing antibodies against one or more DENV serotypes, which were supposed to be maternally transferred antibodies. DENV-1 was the most prevalent, followed by DENV-3, DENV-2, and DENV-4 [14]. Accordingly, a recent study has investigated dengue antibodies seroprevalence among mothers living in Ban Pong, Ratchaburi Province. The results showed that 97% of mothers had dengue neutralizing antibodies to at least one dengue serotype in their sera. Moreover, the proportion of cord sera with dengue neutralizing antibodies to all four dengue serotypes was high at birth and similar to their mother's sera [15]. Based on these results, the antibodies may have been of maternal origin. In older children, the antibodies might be the result of exposure to infected mosquitoes.

As there is an increased risk of hospitalization [16] and severe dengue in seronegative individuals, the WHO SAGE committee recommended using Dengvaxia only in scenes with seroprevalence of approximately 70% or more at the age of 9 years [17]. Our data found that the seropositive rate among 10-14 age groups was 68%. This indicated that such vaccination programs might not be safe and effective in the Thai population. When tetravalent dengue vaccines become available for use in dengue-endemic countries, it should be carefully considered whether to target children who are nine years old or older than that.

This study also shows that both RDT and ELISA have very high specificity for identifying PDI. The RDT demonstrated low sensitivity (35%), while the ELISA displayed greater sensitivity (87%). Both RDTs and ELISA are widely used for the diagnosis of DENV infection [18]. Dengue RDTs are convenient for surveillance in resource-limited settings since they require fewer essential laboratory equipment. Currently, no study evaluated the performance of RDT (SD Bioline) for the past DENV infection. A recent systematic review indicated that the sensitivity and specificity of dengue IgG RDTs were typically above 75% and 80%, respectively, compared with IgG ELISA testing in acute secondary DENV infections and convalescent time points after recent illness [19].

Bonaparte M. et al. evaluated the performance of RDT (Biocan Diagnostics Inc) and ELISA (SciMedx) in identifying individuals with PDI in Puerto Rico, using the PRNT50 as the reference test. Both RDT and ELISA showed high specificity (>99%) but moderate sensitivities (61 and 76%, respectively) [20]. However, in our evaluation, the RDT showed a disappointing sensitivity compared to ELISA. Based on the present study, upon applying the RDT to screen PDI, almost 65% of those supposed to be seropositive will be classified as a seronegative group. Eventually, they will miss the opportunity for dengue vaccination. This suggests that the dengue IgG RDT (SD Bioline) may not appropriate for identifying PDI, especially in the endemic areas. However, the desirable characteristics of dengue pre-vaccination screening tests have been debated [21, 22]. It has recently been recommended that the desired sensitivity and specificity to be at least 90% [23]. Recently, there is a newly developed, automated immunoglobulin fluorescence immunoassay for determining dengue serostatus. However, its specificity and sensitivity are still insufficient for pre-vaccination screening [24].

IgG ELISA measures total anti-DENV binding antibodies, whereas the PRNT measures the neutralization capacity of the antibody response [6], which would be a part of binding antibody and more specific to a particular virus than ELISA. The IgG ELISA might be more sensitive and less specific as it also detects non-neutralizing and cross-reactive antibodies to other flaviviruses within a serum specimen [25]. However, we found that those who were tested positive for dengue IgG ELISA also were tested positive for PRNT, which would minimize the likelihood of false positives in settings with past or current co-circulation with other flaviviruses. Doum D. et al. reported that the IgG ELISA optical densities (OD) with age were consistently higher in rural settings (R^2=0.46-0.54) and lower in rural settings (R^2=0.15-0.28) [11]. Our urban study indicated that the IgG ELISA titers (RU/ml) with age were low

(R^2=0.14). Moreover, we found a positive correlation between IgG ELISA titers and NT50 titers.

There are, however, several limitations in our study. First, the sample collection was limited to the Ratchaburi province, which may not reflect the whole population. Second, children under ten years old were not available in this study. Hence, we could not determine the seroprevalence among 9-year-old children, which was also the targeted age for vaccination. Third, we tested only one RDT (SD Bioline) and one ELISA kit (EUROIMMUN). It should be noted that the sensitivity for identifying PDI may vary among different companies. Despite these limitations, this study provides the most recent seroprevalence data for dengue viruses from the western part of Thailand. We performed an age-stratified serosurvey using PRNT50, distinguishing specific DENV serotypes and representing the true incidence of immunity to DENV. Moreover, the RDT and ELISA performance in identifying PDI was demonstrated, which might be used to determine their suitability for pre-vaccination screening.

Conclusions

In conclusion, this study confirms the distribution of multiple dengue serotypes in Thailand. The frequency of DENV infection was increased in a time-dependent manner as the high prevalence of multitypic DENV serotypes was observed in the older age group. Based on the performance evaluation indicated that, if we use the RDT to screen PDI, almost 65% of those supposed to be seropositive will miss the opportunity for dengue vaccination. Anti-Dengue IgG ELISA is more suitable than the RTD for vaccine introduction and identifying which populations to target.

Acknowledgments

We are grateful to the volunteer children and their parents for participating in the study. We thank the medical staff at Banpong hospital and Potharam hospital for their support of this project. We also thank Stefan Fernandez and the laboratory staff of the Department of Virology, Armed Forces Research Institute of Medical Sciences (Bangkok, Thailand) for PRNT assay training and reagents.

Reference:

1. Lanciotti RS, Gubler DJ, Trent DW. Molecular evolution and phylogeny of dengue-4 viruses. J Gen Virol. 1997;78 (9):2279-84. Epub 1997/09/18. doi: 10.1099/0022-1317-78-9-2279. PubMed PMID: 9292015.

2. Rigau-Pérez JG, Clark GG, Gubler DJ, Reiter P, Sanders EJ, Vorndam AV. Dengue and dengue haemorrhagic fever. Lancet. 1998;352(9132):971-7. Epub 1998/09/30. doi: 10.1016/s0140-6736(97)12483-7. PubMed PMID: 9752834.

3. Sridhar S, Luedtke A, Langevin E, Zhu M, Bonaparte M, Machabert T, et al. Effect of Dengue Serostatus on Dengue Vaccine Safety and Efficacy. N Engl J Med. 2018;379(4):327-40. Epub 2018/06/14. doi: 10.1056/NEJMoa1800820. PubMed PMID: 29897841.

4. World Health O. Dengue vaccine: WHO position paper, July 2016 - recommendations. Vaccine. 2017;35(9):1200-1. Epub 2017/02/12. doi: 10.1016/j.vaccine.2016.10.070. PubMed PMID: 28185744.

5. Imrie A, Meeks J, Gurary A, Sukhbaatar M, Truong TT, Cropp CB, et al. Antibody to dengue 1 detected more than 60 years after infection. Viral Immunol. 2007;20(4):672-5. Epub 2007/12/27. doi: 10.1089/vim.2007.0050. PubMed PMID: 18158740; PubMed Central PMCID: PMCPMC3084288.

6. Russell PK, Nisalak A, Sukhavachana P, Vivona S. A plaque reduction test for dengue virus neutralizing antibodies. J Immunol. 1967;99(2):285-90. Epub 1967/08/01. PubMed PMID: 6031202.

7. Roehrig JT, Hombach J, Barrett AD. Guidelines for Plaque-Reduction Neutralization Testing of Human Antibodies to Dengue Viruses. Viral Immunol. 2008;21(2):123-32. Epub 2008/05/15. doi: 10.1089/vim.2008.0007. PubMed PMID: 18476771.

8. Peeling RW, Artsob H, Pelegrino JL, Buchy P, Cardosa MJ, Devi S, et al. Evaluation of diagnostic tests: dengue. Nat Rev Microbiol. 2010;8(12 Suppl):S30-8. Epub 2011/05/07. doi: 10.1038/nrmicro2459. PubMed PMID: 21548185.

9. Malhotra RK, Indrayan A. A simple nomogram for sample size for estimating sensitivity and specificity of medical tests. Indian J Ophthalmol. 2010;58(6):519-22. Epub 2010/10/19. doi: 10.4103/0301-4738.71699. PubMed PMID: 20952837; PubMed Central PMCID: PMCPMC2993983.

10. Vongpunsawad S, Intharasongkroh D, Thongmee T, Poovorawan Y. Seroprevalence of antibodies to dengue and chikungunya viruses in Thailand. PLoS One. 2017;12(6):e0180560. Epub 2017/07/01. doi: 10.1371/journal.pone.0180560. PubMed PMID: 28662144; PubMed Central PMCID: PMCPMC5491253.

11. Doum D, Overgaard HJ, Mayxay M, Suttiprapa S, Saichua P, Ekalaksananan T, et al. Correction: Doum, D., et al. Dengue Seroprevalence and Seroconversion in Urban and Rural Populations in Northeastern Thailand and Southern Laos. Int. J. Environ. Res. Public Health 2020, 17, 9134. Int J Environ Res Public Health. 2021;18(4). Epub 2021/02/10. doi: 10.3390/ijerph18041439. PubMed PMID: 33557444; PubMed Central PMCID: PMCPMC7913739.

12. Endy TP, Nisalak A, Chunsuttitwat S, Vaughn DW, Green S, Ennis FA, et al. Relationship of preexisting dengue virus (DV) neutralizing antibody levels to viremia and

severity of disease in a prospective cohort study of DV infection in Thailand. J Infect Dis. 2004;189(6):990-1000. Epub 2004/03/05. doi: 10.1086/382280. PubMed PMID: 14999601.

13. Jang WS, Kwak SY, May WL, Yang DJ, Nam J, Lim CS. Comparative evaluation of three dengue duo rapid test kits to detect NS1, IgM, and IgG associated with acute dengue in children in Myanmar. PLoS One. 2019;14(3):e0213451. Epub 2019/03/14. doi: 10.1371/journal.pone.0213451. PubMed PMID: 30865680; PubMed Central PMCID: PMCPMC6415848.

14. Pengsaa K, Limkittikul K, Luxemburger C, Yoksan S, Chambonneau L, Ariyasriwatana C, et al. Age-specific prevalence of dengue antibodies in Bangkok infants and children. Pediatr Infect Dis J. 2008;27(5):461-3. Epub 2008/03/25. doi: 10.1097/INF.0b013e3181646d45. PubMed PMID: 18360303.

15. Khamim K, Hattasingh W, Nisalak A, Kaewkungwal J, Fernandez S, Thaisomboonsuk B, et al. Neutralizing dengue antibody in pregnant Thai women and cord blood. PLoS Negl Trop Dis. 2015;9(2):e0003396. Epub 2015/02/07. doi: 10.1371/journal.pntd.0003396. PubMed PMID: 25658481; PubMed Central PMCID: PMCPMC4320096.

16. Hadinegoro SR, Arredondo-García JL, Capeding MR, Deseda C, Chotpitayasunondh T, Dietze R, et al. Efficacy and Long-Term Safety of a Dengue Vaccine in Regions of Endemic Disease. N Engl J Med. 2015;373(13):1195-206. Epub 2015/07/28. doi: 10.1056/NEJMoa1506223. PubMed PMID: 26214039.

17. Dengue vaccine: WHO position paper, September 2018 - Recommendations. Vaccine. 2019;37(35):4848-9. Epub 2018/11/15. doi: 10.1016/j.vaccine.2018.09.063. PubMed PMID: 30424888.

18. Raafat N, Blacksell SD, Maude RJ. A review of dengue diagnostics and implications for surveillance and control. Trans R Soc Trop Med Hyg. 2019;113(11):653-60. Epub

2019/08/01. doi: 10.1093/trstmh/trz068. PubMed PMID: 31365115; PubMed Central PMCID: PMCPMC6836713.

19. Luo R, Fongwen N, Kelly-Cirino C, Harris E, Wilder-Smith A, Peeling RW. Rapid diagnostic tests for determining dengue serostatus: a systematic review and key informant interviews. Clin Microbiol Infect. 2019;25(6):659-66. Epub 2019/01/22. doi: 10.1016/j.cmi.2019.01.002. PubMed PMID: 30664935; PubMed Central PMCID: PMCPMC6543064.

20. Bonaparte M, Huleatt J, Hodge S, Zheng L, Lustig Y, DiazGranados CA, et al. Evaluation of dengue serological tests available in Puerto Rico for identification of prior dengue infection for prevaccination screening. Diagn Microbiol Infect Dis. 2020;96(3):114918. Epub 2019/12/17. doi: 10.1016/j.diagmicrobio.2019.114918. PubMed PMID: 31839333.

21. Rodríguez-Barraquer I, Salje H, Cummings DA. Dengue pre-vaccination screening and positive predictive values. Lancet Infect Dis. 2019;19(2):132-4. Epub 2019/02/05. doi: 10.1016/s1473-3099(18)30799-0. PubMed PMID: 30712834.

22. Flasche S, Smith PG. Sensitivity and negative predictive value for a rapid dengue test. Lancet Infect Dis. 2019;19(5):465-6. Epub 2019/04/30. doi: 10.1016/s1473-3099(19)30167-7. PubMed PMID: 31034385.

23. Wilder-Smith A, Smith PG, Luo R, Kelly-Cirino C, Curry D, Larson H, et al. Pre-vaccination screening strategies for the use of the CYD-TDV dengue vaccine: A meeting report. Vaccine. 2019;37(36):5137-46. Epub 2019/08/05. doi: 10.1016/j.vaccine.2019.07.016. PubMed PMID: 31377079.

24. Daag JV, Ylade M, Adams C, Jadi R, Crisostomo MV, Alpay R, et al. Evaluation of a new point-of-care test to determine prior dengue infection for potential use in pre-vaccination

screening. Clin Microbiol Infect. 2020. Epub 2020/09/01. doi: 10.1016/j.cmi.2020.08.026. PubMed PMID: 32866651.

25. WHO Guidelines Approved by the Guidelines Review Committee. Dengue: Guidelines for Diagnosis, Treatment, Prevention and Control: New Edition. Geneva: World Health Organization. 2009.

Figure 1

Click here to access/download;Figure;Fig1.tif

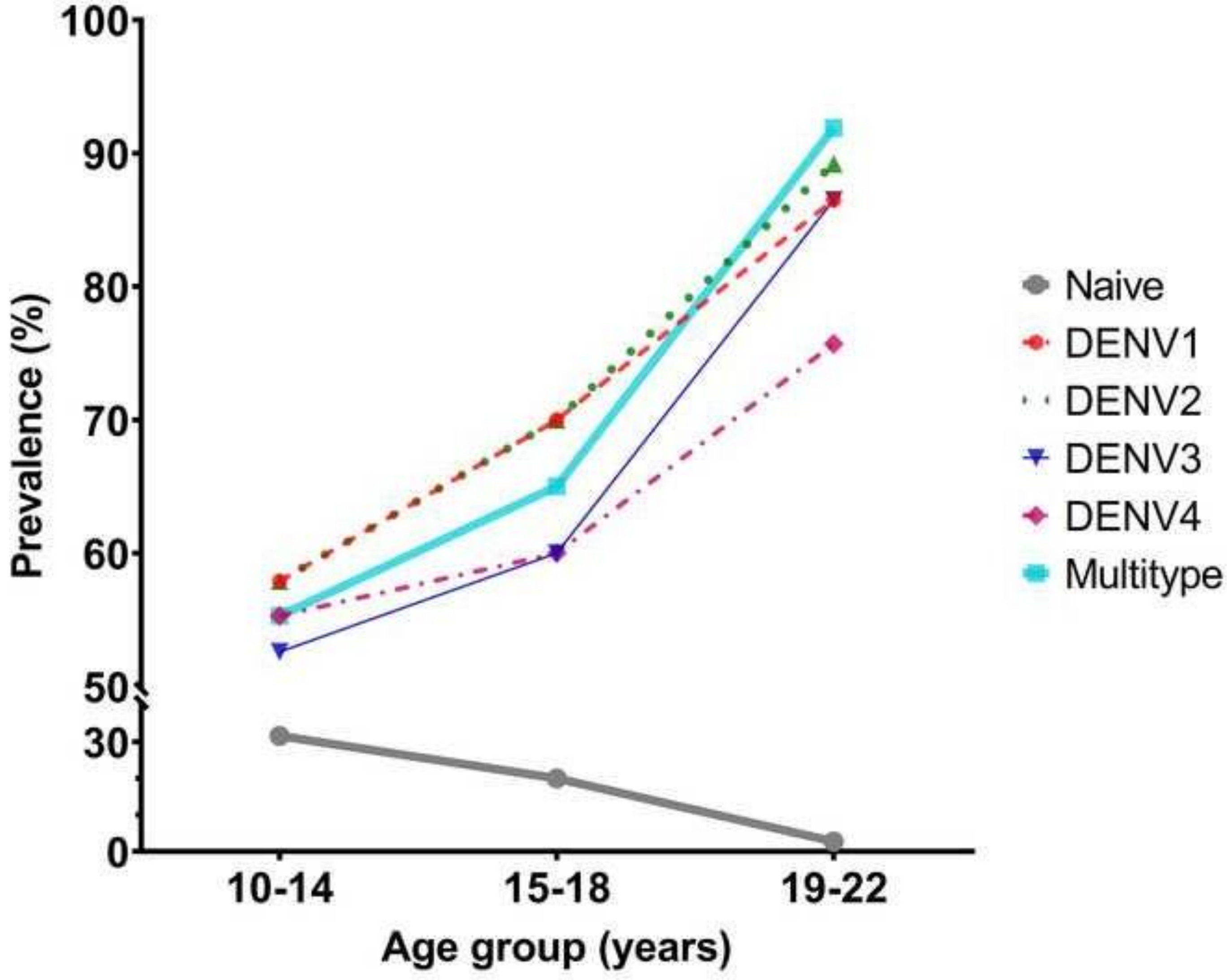

Figure 2

Click here to access/download;Figure;Fig1.tif

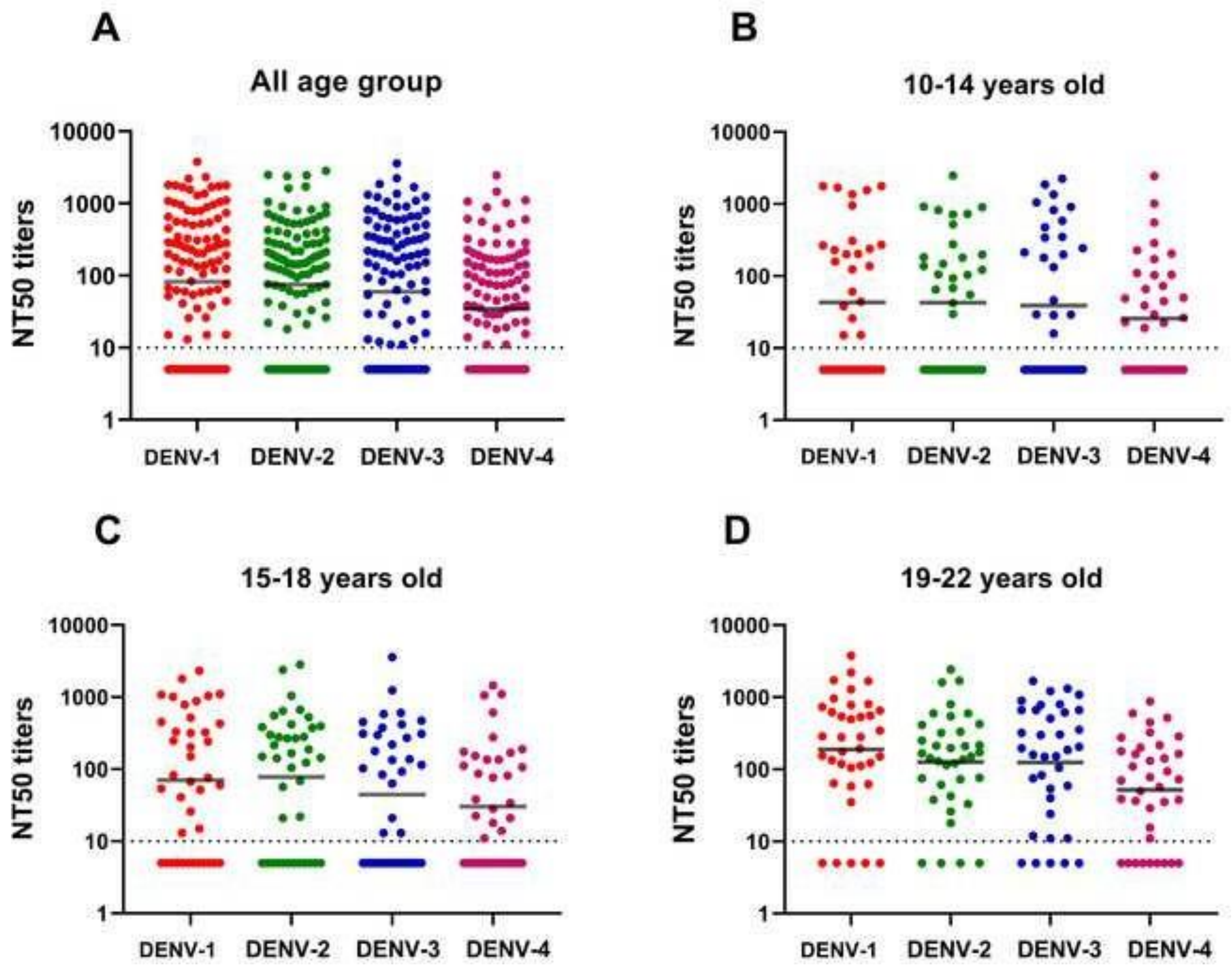

Figure 3

Click here to access/download;Figure;Fig1.tif

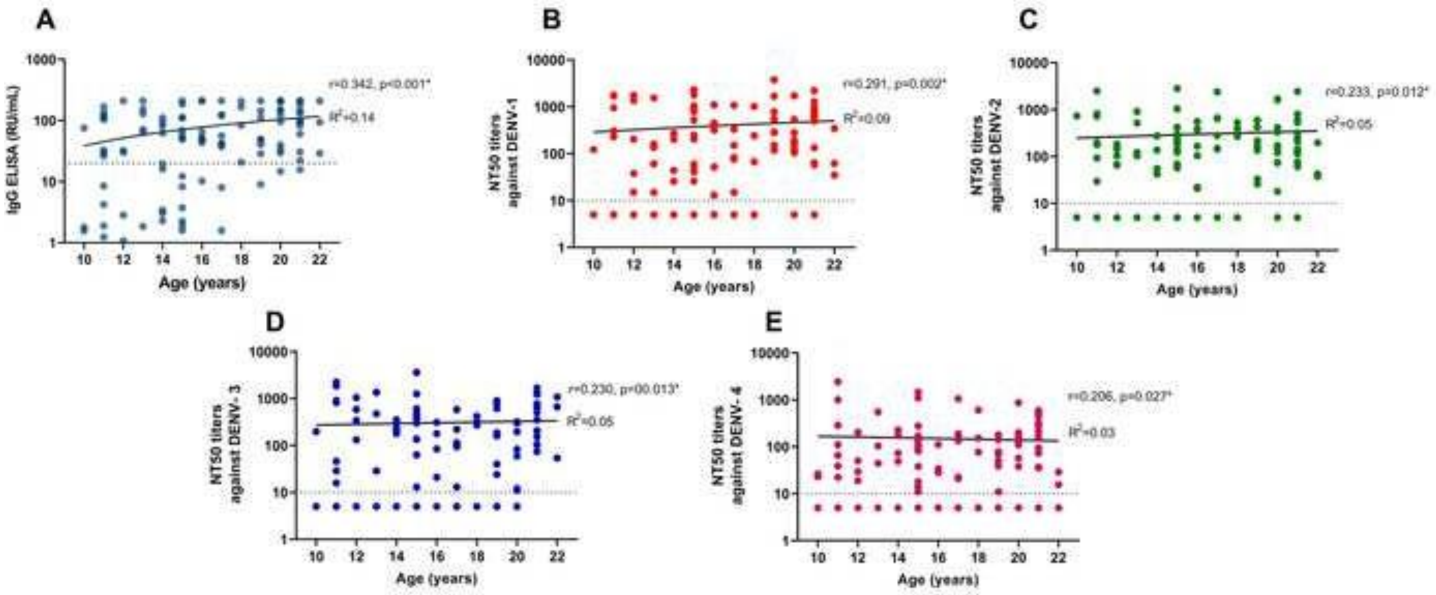

STROBE checklist

Click here to access/download
Supporting Information
STROBEchecklist1.doc
