# Supplementary material for: Dengue pre-vaccination screening test evaluation for the use of dengue vaccine in an endemic area
Source: PLoS One. 2021 Sep 10;16(9):e0257182. doi: 10.1371/journal.pone.0257182 (PMC8432984; doi:10.1371/journal.pone.0257182)
Supplement: Attachment — Submitted filename: PONE-D-21-09490_reviewer (1).docx [file pone.0257182.s004.docx]

PLOS ONE

**Dengue pre-vaccination screening test evaluation for the use of dengue vaccine in an endemic area**

--Manuscript Draft--

| **Manuscript Number:** | PONE-D-21-09490 |
| --- | --- |
| **Article Type:** | Research Article |
| **Full Title:** | Dengue pre-vaccination screening test evaluation for the use of dengue vaccine in an endemic area |
| **Short Title:** | Dengue pre-vaccination screening test evaluation |
| **Corresponding Author:** | Nattachai Srisawat  King Chulalongkorn Memorial Hospital bangkok, THAILAND |
| **Keywords:** | CYD-TDV; Dengvaxia; Dengue serostatus; Prior dengue infection; Prevaccination screening test; rapid diagnostic test; ELISA; Sensitivity; Specificity |
| **Abstract:** | Background: The dengue vaccine (Dengvaxia) is only recommended in individuals who have had prior dengue infection (PDI). In this study, we aimed to perform a serosurvey to support decision-making for vaccine introduction and identify which populations to target. We also evaluated the performance of the serological tests using plaque reduction neutralization test (PRNT) as a reference test in identifying PDI to determine their suitability for pre-vaccination screening.  Methods: We enrolled 115 healthy individuals between 10 and 22 years of age living in Ratchaburi province, Thailand. The serum samples were tested by PRNT to measure the prevalence and concentration of serotype-specific neutralizing antibodies. The performance of the IgG rapid diagnostic test (RDT, SD Bioline, Korea) and IgG enzyme-linked immunosorbent assay (ELISA, EUROIMMUN, Germany) in identifying PDI were evaluated by using PRNT as a reference method.  Results: Overall, 94 (81.7%) of individuals neutralized one or more dengue serotypes at a titer threshold of equal or more than 10. Multitypic profiles were observed in 70.4% of the samples, which increased to 91.9% in subjects aged 19-22. Amongst monotypic samples, the highest proportion was reactive against DENV-1, followed by DENV-2, DENV-3, and DENV-4. The highest anti-dengue antibody titers were recorded against DENV-1 and increased with age to a geometric mean of 188.6 in the oldest age group. Both RDT and ELISA exhibited 100% specificity. The RDT demonstrated low sensitivity (35%), while the ELISA displayed greater sensitivity (87%).  Conclusions: Almost eighty percent of Thai adolescents and youth had already been exposed to the dengue virus. All four dengue serotypes have been widely circulating in Thailand. The dengue IgG RDTs have shown low sensitivity and might not suitable for dengue pre-vaccination screening. Based on these results, IgG ELISA could safely guide vaccination in Thailand. |
| **Order of Authors:** | Umaporn Limothai |
|  | Sasipha Tachaboon |
|  | Janejira Dinhuzen |
|  | Taweewun Hunsawong |
|  | Prapapun Ong-ajchaowlerd |
|  | Butsaya Thaisomboonsuk |
|  | Supachoke Trongkamolchai |
|  | Mananya Wanpaisitkul |
|  | Chatchai Chulapornsiri |
|  | Anongrat Tiawilai |
|  | Thawat Tiawilai |
|  |  |

*Powered by Editorial Manager® and ProduXion Manager® from Aries Systems Corporation*

|  | Terapong Tantawichien |
| --- | --- |
|  | Usa Thisyakorn |
|  | Nattachai Srisawat |
| **Additional Information:** |  |
| **Question** | **Response** |
| **Financial Disclosure**  Enter a financial disclosure statement that describes the sources of funding for the work included in this submission. Review the [submission guidelines](http://journals.plos.org/plosone/s/submission-guidelines#loc-financial-disclosure-statement) for detailed requirements. View published research articles from [*PLOS ONE*](http://journals.plos.org/plosone/) for specific examples.  This statement is required for submission and **will appear in the published article** if the submission is accepted. Please make sure it is accurate. | Umaporn Limothhai received funding from the Second Century Fund (C2F), Chulalongkorn University.  Nattachai Srisawat received funding from the Jongkolneenithi foundation,  the Medical Association of Thailand, the Tropical Medicine Cluster, Chulalongkorn University and Ratchadaphiseksomphot Endowment Fund, Chulalongkorn University. The funders had no role in study design, data collection and analysis, decision to publish, or preparation of the manuscript. |
| **Unfunded studies**  Enter: *The author(s) received no specific funding for this work.*  **Funded studies**  Enter a statement with the following details:   - Initials of the authors who received each award - Grant numbers awarded to each author - The full name of each funder - URL of each funder website - Did the sponsors or funders play any role in the study design, data collection and analysis, decision to publish, or preparation of the manuscript? - **NO** - Include this sentence at the end of your statement: *The funders had no role in study design, data collection and analysis, decision to publish, or preparation of the manuscript.* - **YES** - Specify the role(s) played. |  |
| * typeset |  |
| **Competing Interests**  Use the instructions below to enter a competing interest statement for this submission. On behalf of all authors, disclose any [competing interests](http://journals.plos.org/plosone/s/competing-interests) that could be perceived to bias this | The authors have declared that no competing interests exist. |

*Powered by Editorial Manager® and ProduXion Manager® from Aries Systems Corporation*

| work—acknowledging all financial support and any other relevant financial or non- financial competing interests.  This statement is required for submission and **will appear in the published article** if the submission is accepted. Please make sure it is accurate and that any funding sources listed in your Funding Information later in the submission form are also declared in your Financial Disclosure statement.  View published research articles from  [*PLOS ONE*](http://journals.plos.org/plosone/) for specific examples. |  |
| --- | --- |
| **NO authors have competing interests**  Enter: *The authors have declared that no competing interests exist.*  **Authors with competing interests**  Enter competing interest details beginning with this statement:  *I have read the journal's policy and the authors of this manuscript have the following competing interests: [insert competing interests here]* |  |
| * typeset |  |
| **Ethics Statement**  Enter an ethics statement for this submission. This statement is required if the study involved:   - Human participants - Human specimens or tissue - Vertebrate animals or cephalopods - Vertebrate embryos or tissues - Field research   Write "N/A" if the submission does not require an ethics statement. | The study was conducted according to the Helsinki Declaration and Good Clinical Practice guidelines. The study protocol was approved by the ethical committee of Banpong hospital (REC 008/2562) and Potharam hospital (REC 31/2562). The written informed consent was obtained from subjects ≥18 years old and parents of subjects  <18 years old. |

*Powered by Editorial Manager® and ProduXion Manager® from Aries Systems Corporation*

| General guidance is provided below. Consult the [submission guidelines](http://journals.plos.org/plosone/s/submission-guidelines#loc-guidelines-for-specific-study-types) for detailed instructions. **Make sure that all information entered here is included in the Methods section of the manuscript.** |  |
| --- | --- |
| **Format for specific study types**  **Human Subject Research (involving human participants and/or tissue)**   - Give the name of the institutional review board or ethics committee that approved the study - Include the approval number and/or a statement indicating approval of this research - Indicate the form of consent obtained (written/oral) or the reason that consent was not obtained (e.g. the data were analyzed anonymously)   **Animal Research (involving vertebrate animals, embryos or tissues)**   - Provide the name of the Institutional Animal Care and Use Committee (IACUC) or other relevant ethics board that reviewed the study protocol, and indicate whether they approved this research or granted a formal waiver of ethical approval - Include an approval number if one was obtained - If the study involved *non-human primates*, add *additional details* about animal welfare and steps taken to ameliorate suffering - If anesthesia, euthanasia, or any kind of animal sacrifice is part of the study, include briefly which substances and/or methods were applied   **Field Research**  Include the following details if this study involves the collection of plant, animal, or other materials from a natural setting:   - Field permit number - Name of the institution or relevant body that granted permission |  |
|  |  |
| **Data Availability**  Authors are required to make all data underlying the findings described fully | Yes - all data are fully available without restriction |

*Powered by Editorial Manager® and ProduXion Manager® from Aries Systems Corporation*

| available, without restriction, and from the time of publication. PLOS allows rare exceptions to address legal and ethical concerns. See the [PLOS Data Policy](http://journals.plos.org/plosone/s/data-availability) and [FAQ](http://journals.plos.org/plosone/s/data-availability#loc-faqs-for-data-policy) for detailed information.  A Data Availability Statement describing where the data can be found is required at submission. Your answers to this question constitute the Data Availability Statement and **will be published in the article**, if accepted. |  |
| --- | --- |
| **Important:** Stating ‘data available on request from the author’ is not sufficient. If your data are only available upon request, select ‘No’ for the first question and explain your exceptional situation in the text box. |  |
| Do the authors confirm that all data underlying the findings described in their manuscript are fully available without restriction? |  |
| **Describe where the data may be found in full sentences. If you are copying our sample text, replace any instances of XXX with the appropriate details.**   - If the data are **held or will be held in a public repository**, include URLs, accession numbers or DOIs. If this information will only be available after acceptance, indicate this by ticking the box below. For example: *All XXX files are available from the XXX database (accession number(s) XXX, XXX.*). - If the data are all contained **within the manuscript and/or Supporting Information files**, enter the following: *All relevant data are within the manuscript and its Supporting Information files.* - If neither of these applies but you are able to provide **details of access elsewhere**, with or without limitations, please do so. For example:   *Data cannot be shared publicly because of [XXX]. Data are available from the XXX Institutional Data Access / Ethics* | All relevant data are within the manuscript and its Supporting Information files. |

*Powered by Editorial Manager® and ProduXion Manager® from Aries Systems Corporation*

| *Committee (contact via XXX) for researchers who meet the criteria for access to confidential data.*  *The data underlying the results presented in the study are available from (include the name of the third party and contact information or URL).*   - This text is appropriate if the data are owned by a third party and authors do not have permission to share the data.   * typeset |  |
| --- | --- |
| Additional data availability information: |  |

*Powered by Editorial Manager® and ProduXion Manager® from Aries Systems Corporation*

1

# 1 Dengue pre-vaccination screening test evaluation for the use of dengue vaccine in an

2 **endemic area**

3

4 Umaporn Limothai^1,2^, Sasipha Tachaboon^1,2,^ Janejira Dinhuzen^1,2^, Taweewun Hunsawong^3^,
5 Prapapun Ong-ajchaowlerd^3^, Butsaya	Thaisomboonsuk^3^, Supachoke	Trongkamolchai^4^,
6 Mananya Wanpaisitkul^4^, Chatchai Chulapornsiri^4^, Anongrat Tiawilai^5^, Thawat Tiawilai^5^,
7 Terapong Tantawichien^6,7^, Usa Thisyakorn^7^, Nattachai Srisawat^1,2,7-10*^ 8

9	1. Excellence Center for Critical Care Nephrology, King Chulalongkorn Memorial Hospital,

10 Bangkok, Thailand.
11 2. Critical Care Nephrology Research Unit, Chulalongkorn University, Bangkok, Thailand.
12 3. Department of Virology, Armed Forces Research Institute of Medical Sciences, Bangkok,
13 Thailand.
14 4. Banpong Hospital, Ratchaburi, Thailand.
15 5. Photharam Hospital, Ratchaburi, Thailand.
16 6. Division of Infectious Diseases, Department of Medicine, Faculty of Medicine,
17 Chulalongkorn University, Bangkok, Thailand.
18 7. Tropical Medicine Cluster, Chulalongkorn University, Bangkok, Thailand.
19 8. Division of	Nephrology, Department	of	Medicine, Faculty	of	Medicine, King
20 Chulalongkorn Memorial Hospital, Bangkok 10330, Thailand.
21 9. Center for Critical Care Nephrology, The CRISMA Center, Department of Critical Care
22 Medicine, University of Pittsburgh, School of Medicine, Pittsburgh, PA, USA.
23 10. Academy of Science, Royal Society of Thailand, Bangkok, Thailand.

24

25

26 **Keyword:** CYD-TDV; Dengvaxia; Dengue serostatus; Prior dengue infection
27 Prevaccination screening test; rapid diagnostic test; ELISA; Sensitivity; Specificity 28

29	**Short title:** Dengue pre-vaccination screening test evaluation 30

31 ***Corresponding Author:** Assoc. Prof. Dr. Nattachai Srisawat
32 Excellence Center for Critical Care Nephrology, Thai Red Cross Society, King Chulalongkorn
33 Memorial Hospital
34 1873, Rama 4 Rd., Lumphini, Pathumwan, Bangkok 10330
35 Phone (662) – 2564000 Ext.3597 Email: [drnattachai@yahoo.com](mailto:drnattachai@yahoo.com)

# 36 Abstract

37 **Background:** The dengue vaccine (Dengvaxia) is only recommended in individuals who have
38 had prior dengue infection (PDI). In this study, we aimed to perform a serosurvey to support
39 decision-making for vaccine introduction and identify which populations to target. We also
40 evaluated the performance of the serological tests using plaque reduction neutralization test
41 (PRNT) as a reference test in identifying PDI to determine their suitability for pre-vaccination
42 screening.
43 **Methods:** We enrolled 115 healthy individuals between 10 and 22 years of age living in
44 Ratchaburi province, Thailand. The serum samples were tested by PRNT to measure the
45 prevalence and concentration of serotype-specific neutralizing antibodies. The performance of
46 the IgG	rapid	diagnostic	test	(RDT, SD	Bioline, Korea)	and	IgG	enzyme-linked
47 immunosorbent assay (ELISA, EUROIMMUN, Germany) in identifying PDI were evaluated
48 by using PRNT as a reference method.
49 **Results:** Overall, 94 (81.7%) of individuals neutralized one or more dengue serotypes at a titer
50 threshold of equal or more than 10. Multitypic profiles were observed in 70.4% of the samples,
51 which increased to 91.9% in subjects aged 19-22. Amongst monotypic samples, the highest
52 proportion was reactive against DENV-1, followed by DENV-2, DENV-3, and DENV-4. The
53 highest anti-dengue antibody titers were recorded against DENV-1 and increased with age to
54 a geometric mean of 188.6 in the oldest age group. Both RDT and ELISA exhibited 100%
55 specificity. The RDT demonstrated low sensitivity (35%), while the ELISA displayed greater
56 sensitivity (87%).
57 **Conclusions:** Almost eighty percent of Thai adolescents and youth had already been exposed
58 to the dengue virus. All four dengue serotypes have been widely circulating in Thailand. The
59 dengue IgG RDTs have shown low sensitivity and might not suitable for dengue pre-
60 vaccination screening. Based on these results, IgG ELISA could safely guide vaccination in
61 Thailand.

62

# 63 Introduction

64 Dengue is a mosquito-borne viral disease that has rapidly spread across the tropical and
65 subtropical regions. Dengue virus consists of four serotypes with antigenic differences (DENV-
66 1 to 4) [1]. It has a broad clinical spectrum that includes both severe and non-severe clinical
67 manifestations [2]. The first dengue vaccine, a live, attenuated, tetravalent dengue vaccine
68 (Dengvaxia or CYD-TDV) developed by Sanofi Pasteur, was licensed and available in some
69 countries. The efficacy trials showed that Dengvaxia protected against severe dengue in people
70 who had exposure to dengue prior to vaccination. However, there was evidence of a higher
71 longer-term risk of severe dengue in vaccinated persons who had not been exposed to dengue
72 [3]. World Health Organization (WHO) Strategic Advisory Group of Experts (SAGE)
73 recommends using Dengvaxia in children 9-16 years old living in highly dengue-endemic areas
74 . Furthermore, in defining people to be targeted for vaccination, prior dengue infection
75 (PDI), as measured by seroprevalence, should be approximately 70% or more [4]. To minimize
76 potentially harmful effects, we should ensure that the age group targeted for vaccination has
77 enough pre-existing dengue immunity to benefit from the vaccine (add reference).
78 Age-stratified serosurveys are currently the best method for selecting populations
79 appropriate for vaccination. Immunoglobulin G (IgG)-class antibodies to dengue are useful
80 serological markers for PDI as they can persist over an individual's lifetime [5]. Plaque
81 reduction neutralization test (PRNT) is the most specific serological test for determining type-
82 specific neutralizing antibodies to an infecting virus. This technique can be used to confirm the presence of the neutralization antibodies against Flaviviruses in the serum samples. ~~This technique can be used to confirm the~~
83 ~~infecting flavivirus from a convalescent serum sample~~ [6, 7]. However, PRNT is a labor-
84 intensive, relatively costly test, and it might not be convenient for routine surveillance
85 purposes. Commercial IgG rapid diagnostic test (RDT) and ~~an~~ IgG enzyme-linked immunosorbent assay (ELISA) are commonly used to diagnose acute dengue infection [8]. Nonetheless, the performance data for these tests for the identification of PDI are still limited (reference). Explain more/provide some data from previous studies.
86 In this study, we aimed to perform a comparative serosurvey to support decision-making for dengue
87 vaccine introduction in ……. ages. We also evaluated the performance of the serological tests as a pre-
88 screening test in identifying PDI to determine their suitability for pre-vaccination screening in
89 Thailand.

92

# 93 Materials and methods

94 **Ethics Statement**
95 The study was conducted according to the Helsinki Declaration and Good Clinical
96 Practice guidelines. The study protocol was approved by the ethical committee of Banpong
97 hospital (REC 008/2562) and Potharam hospital (REC 31/2562). The written informed consent
98 was obtained from subjects ≥18 years old and parents of subjects <18 years old.
99 **Cells and Viruses**
100 In this section, you must add all cells and viruses that you have used in this experiment including the passage numbers, source of the cells and viruses.

# 101 Study Location and Population

102 The study was conducted in Banpong and Potharam district, Ratchaburi province, an
103 urban center located in the western part of Thailand. Healthy children (you must include the criteria here, how do you considered them healthy?) between 9 and 22 years
104 of age at the enrollment and residing in Banpong or Potharam district were eligible to
105 participate. Using the sample size calculation by Malhotra RK. and Indrayan A. [9], assuming
106 91% seroprevalence [10, 11], expected sensitivity and specificity of 95% and 95%,
107 respectively, and desired precision of 0.05, we estimate that at least 80 samples would need to
108 be tested. With indeterminate and invalid results, we tested 115 samples. Specimens were
109 collected from August 2019-August 2020. The blood samples were collected in anticoagulant-
110 free vacutainer tubes. The sera were separated and maintained refrigerated at 2-8˚C. The
111 specimens were then transported on the day of collection to the central laboratory at
112 Chulalongkorn hospital and stored at -80°C before testing.

# 113 Plaque Reduction Neutralization Test (PRNT)

# 114 Please add the name of companies that you purchased all the materials from them.

115 PRNT was performed to determine the levels of virus type-specific neutralizing (NT)
116 antibodies using standard methods, as previously described [6, 12]. (Did you heat inactivated the serums before the experiment?) Briefly, serum was diluted in a four-fold serial
117 dilution starting from 1:10 to 1:2,560 and separately mixed with an equal volume of each
118 DENV serotype (Which viruses, name, culture history, passage number, which moi. Are these viruses circulating in your country?????) before incubation in a 35^o^C water bath for 1 hour. The mixture was then
119 inoculated into Rhesus monkey kidney epithelial cells (LLC-MK2 cells) in duplicate wells of
120 a 12-well plate and incubated at room temperature for 1 hour on a rocker platform. After
121 removing of excess volume of inoculum, washed by???, the first overlay medium (which media and how much?) containing 5% heat-
122 inactivated fetal bovine serum (company name?) in 0.9% low melting point agarose gel (LMP, Ultra PureTM
123 LMP agarose, Invitrogen, USA) was added and incubated for 4-6 days in a 5% CO2 incubator
124 at 35^o^C before staining with 4% neutral red (Sigma, USA) in a second overlay medium (how much) with
125 LMP and incubated for ????. The number of plaques was determined by manual counting. PRNT data were expressed
126 as the reciprocal of the serum dilution causing 50% plaque reduction (NT50) as extrapolated
127 by probit regression which was identified as the highest dilution that can reduce the number of
128 plaques by 50% as compared to the number of plaques in the virus control well. Antibody titers
129 ≥10 were considered positive for virus-neutralizing activity (Could you please explain it more? I think the calculation of PRNT is not correct. You can follow the instruction in this article to do the analysis again: doi: 10.4269/ajtmh.2009.08-0625. **This is my main concern in this manuscript??????????????**
130 An individual who has antibody titers <10 for the four serotypes was classified as naïve. An individual who has NT50 antibody
131 titers ≥10 to only one serotype (no NT titer to other serotypes) was classified as monotypic.
132 Correspondingly, an individual who has NT50 antibody titers ≥10 for more than one serotype
133 was classified as multitypic. You need to explain more about the positive and control in PRNT assay. You must determine a minimum number of plaques in positive control.

# 134 Dengue duo rapid test kit

# 135 Did you heat inactivate the serums?

136 Dengue nonstructural protein 1 (NS1) antigen and dengue IgG/IgM antibody were
137 examined at the enrollment using SD Bioline Dengue Duo NS1 Ag & IgG/IgM (catalog
138 number 11FK46, SD Bioline, Korea) according to the manufacturer's instructions. For the NS1
139 antigen, 100 μL of diluted or undiluted ???serum was added to the left sample well, incubated at RT?????????? and the results were read within
140 15-20 minutes. For IgG/IgM, 10 μL of serum was added to the right sample well, and four
141 drops (90-120 μL) of assay diluent were added to the round assay diluent well. The results were
142 read 15-20 minutes later. Each test included negative and positive????????? control and a test line. The appearance of both the
143 control and test lines indicated a positive result. In contrast, the appearance of only the control
144 line indicated a negative result [13].
145 You must describe how to determine the positivity and negativity in this experiment clearly. There are some good articles in this field. Just an example: doi: 10.4269/ajtmh.2010.10-0117

# 146 Dengue IgG ELISA

147 Anti-Dengue IgG	antibodies	were	assessed	using	commercial	enzyme-linked
148 immunosorbent assay (catalog number EI266b-9601G, EUROIMMUN, Lubeck, Germany)
149 according to the manufacturer’s instructions. Briefly, serum samples were diluted at 1:101, and
150 100 μL of the mixture was then added to the 96-well microplates coated with highly purified DENV-
151 2 particles. Please mention that this kit can detect DENV 1 to 4 and this kit used conserved region. After incubating for 60 minutes at room temperature???????, the wells were washed by????? How many????????, and 100 μL of enzyme
152 conjugate (peroxidase-labeled anti-human IgG) was added to each well. After incubating for
153 30 minutes at RT?????????, the plates were washed by????? How many????????,, and the conjugated complex was visualized by adding 100
154 μL of TMB solution to each well. After incubating for 15 minutes at RT??????, the reaction was stopped by
155 adding 100 μL of stop solution to each well. The colorimetric output was evaluated in a microtiter plate
156 reader at 450 nm. Positive determinations were made against the provided IgG reference sera
157 (cutoff calibrators). Antibody values > 20 relative units/mL were considered seropositive (Can you explain more about this number and how you reached it?).

# 158 Statistical Analysis

159 Descriptive statistics for seropositive rate and other characteristics were performed.
160 Continuous variables are presented as the mean ± standard deviation (SD) in case of a
161 normal distribution and as a median and interquartile range (IQR) in case of non-normally
162 distributed variables. The comparison was performed by one-way ANOVA or Kruskal–Wallis
163 test as appropriate. Categorical variables were presented as numbers with percentages and were
164 compared using the Chi-square test or Fisher’s exact test as appropriate. P-value <0.05 was
165 considered significant. The geometric mean titer and the 95% CI for each age group and dengue
166 serotype were calculated for all samples based on their DENV PRNT results. To calculate the
167 GMT, samples with an antibody titer <10 were given the value of 5. All analyses were
168 performed using IBM SPSS Statistics software version 22.0 (IBM Corp., Armonk, NY)

# 169 Disclaimer

170 Material has been reviewed by the Walter Reed Army Institute of Research. There is
171 no objection to its presentation and/or publication. The opinions or assertions contained herein
172 are the private views of the author, and are not to be construed as official, or as reflecting true

views of the Department of the Army or the Department of Defense.

# 173 Results

174 **Demographic data**
175 Table 1 provides general characteristics of 115 participants included in the study. Their
176 age ranged from 10 to 22 years, with a median of 16 years (IQR 13-20). More than half (67,
177 58.3%) were males. The participants were predominantly (53%) from Ban Pong District.
178 176

**Table 1** Demographic data (any specific reason for these groups?????? Any specific reason for these geographical area?? Is there any available previous data from these regions???)

| **Characteristics** | **Total (N=115)** |
| --- | --- |
| Gender, male, (N, %) | 67 (58.3) |
| Age, years (median, range) | 16 (10-22) |
| 10-14 (N,%) | 38 (33.0) |
| 15-18 (N,%) | 40 (34.8) |
| 19-22 (N,%) | 37 (32.2) |
| Area |  |
| Ban Pong (N,%) | 61 (53.0) |
| Potharam (N,%) | 54 (47.0) |

# 179 Seroprevalence of DENV amongst participants

180 Serological evidence of previous exposure to DENV as denoted by NT50 titers ≥ 10
181 was identified in the serum samples of 94 (81.7%) participants. Seroprevalence did not
182 significantly differ based on gender (p=0.113). To examine the DENV seroprevalence in
183 different age groups, we classified the participant's age into three categories based on school-
184 age, namely 10-14 years, 15-18 years, and 19-22 years (You must mention this in the methods) . The data indicated that increasing age
185 appeared to be associated with increased seropositivity (p=0.005). We also examined the
186 seroprevalence by IgG ELISA and IgG rapid test. The results showed that the overall
187 seropositive rate had decreased to 71.3% by IgG ELISA and 28.7% by IgG rapid test (Table

186	2).

187

194

| 188 | Data are presented as n (%) |
| --- | --- |
| 189 |  |
| 190 | **Neutralizing antibody profile distribution stratified by age** |
| 191 | Samples were categorized according to the NT50 profile. Multitypic profiles were |
| 192 | observed in 70.4% of the subjects, with 55.3% in those aged 10-14 years old, 65.0% in the 15- |
| 193 | 18 years old, and 91.9% in the 19-22 years old (Fig 1). |

**Table 2** Seroprevalence of DENV by NT50 (titers ≥ 10), IgG ELISA, and IgG rapid test (It will be grate to add graph for this data)

| **Characteristics** | **Seropositive**  **by NT50** | **Seropositive**  **by IgG ELISA** | **Seropositive**  **by IgG rapid test** |
| --- | --- | --- | --- |
| **Overall** | 94 (81.7%) | 82 (71.3%) | 33 (28.7%) |
| **Gender** |  |  |  |
| Male | 58 (86.6%) | 50 (74.6%) | 20 (29.9%) |
| Female | 36 (75.0%) | 32 (66.7%) | 13 (27.1%) |
| **Age** |  |  |  |
| 10-14 | 26 (68.4%) | 21 (55.3%) | 10 (26.3%) |
| 15-18 | 32 (80.0%) | 27 (67.5%) | 8 (20.0%) |
| 19-22 | 36 (97.3%) | 34 (91.9%) | 15 (40.5%) |

195 **Fig 1.** Prevalence of individuals with naïve (NT50 titer<10), monotypic (NT50 titer≥10 against
196 to only one dengue serotype), or multitypic (NT50 titer≥10 against more than one serotypes)
197 197
198 198

neutralizing antibody profile, by age group

199 There were 31.6% naïve subjects in the 10-14 years old group, 20.0% of the 15-18 years
200 old group and 2.7% of the 19-22 years old groups, and 18.3% of the overall sample had no
201 detectable neutralizing dengue antibodies at the ten thresholds. (Table 2)
202 Among monotypic samples, the highest proportion of samples were reactive against
203 DENV-2, followed by DENV-1, DENV-3, and DENV-4, a trend observed in the 19-22 years
204 old age groups. The proportion of samples that were reactive against DENV-1 and DENV-2
205 were evenly distributed amongst the 10–14 and 15–18 years old age groups.
206 The distribution of geometric mean NT50 titer (GMT) among the ages 10-22 years old
207 was shown in Fig 2A. DENV-1 had the highest overall GMT (82.2), followed by DENV-2
208 (74.5), DENV-3 (59.3), and DENV-4 (34.2). The trends were also observed in the 10-14 years
209 old age group (Fig 2B, the GMT were 43.1, 42.8, 38.8, and 25.8 respectively), and in the 19-
210 22 years old age group (Fig 2D), the GMT was 188.6, 126.0, 124.9, and 51.7 respectively). For
211 the 15-18 years old age groups (Fig 2C), DENV-2 had the highest GMT (77.7), followed by
212 212
213 213

DENV-1 (70.5), DENV-3 (44.6), and DENV-4 (30.4).

214 **Fig 2.** Dengue serotype-specific geometric mean titer (GMT) by age-group.
215 The long solid line indicates the threshold for seropositive (an NT50 titer ≥10).

# 216 Performance of the dengue IgG rapid test and dengue IgG ELISA for determination of

217 **dengue serostatus**
218 We assessed the performance of the IgG rapid test and IgG ELISA for detection of
219 dengue pre-exposure using the PRNT50 as the reference assay. Of the 94 patients classified as
220 seropositive by PRNT (titers ≥ 10), 61 (64.9%) were tested negative by dengue IgG rapid test,
221 and 12 (12.8%) were tested negative by dengue IgG ELISA. For the dengue IgG rapid test,
222 sensitivity was 35.1%, and specificity was 100%. When we evaluated the dengue IgG ELISA
223 to detect prior dengue infection, specificity was unchanged, but sensitivity increased to 87.2%
224 compared to IgG rapid test. Positive predictive values (PPV) for both tests were equal (100%).
225 Negative predictive values (NPV) were 25.6% and 63.6% for dengue rapid test and IgG
226 226
227 227

ELISA, respectively (Table 3).

228 **Table 3.** Performance of the dengue IgG rapid test and dengue IgG ELISA for dengue
229 serostatus determination using the plaque reduction neutralization test (PRNT) as the reference
230 230
231 231
232 232

assay.

| **Test** | **Dengue rapid test (IgG)** | | **Dengue ELISA (IgG)** | |
| --- | --- | --- | --- | --- |
|  | Positive | Negative | Positive | Negative |
| Positive PRNT (n,%) | 33 (35.1%) | 61 (64.9%) | 82 (87.2%) | 12 (12.8%) |
| Negative PRNT (n,%) | 0 (0.0%) | 21 (100.0%) | 0 (0.0) | 21 (100.0%) |
| Sensitivity | 35.1% | | 87.2% | |
| Specificity | 100.0% | | 100.0% | |
| Positive predictive value | 100.0% | | 100.0% | |
| Negative predictive value | 25.6% | | 63.6% | |

233 The relationship between NT50 titer and dengue IgG level (relative RU/mL by ELISA)
234 and was also examined. The result showed that NT50 titer had a positive correlation with IgG

235	level (r=0.701, p<0.001 for DENV-1, r=0.771, p<0.001 for DENV-2, r=0.667, p<0.001 for

236	DENV-3, and r=0.725, p<0.001 for DENV-4). The distribution of dengue IgG level and NT50

237

238

titer by age was demonstrated in Fig 3.

239 **Fig 3.** Distribution of dengue IgG level (relative units per milliliter by ELISA) and NT50 titer
240 by age
241 The dashed line indicates the threshold for dengue IgG ELISA (20 RU/ml) and NT50 (a titer
242 242
243 243

of 10).

# 244 Discussion

245 We conducted a dengue seroprevalence study, which identified serological evidence
246 for the circulation of all four dengue serotypes among adolescents and youth in Ratchaburi
247 province, Thailand. This study generated data on serotype-specific prevalence in areas where
248 little data were previously available. The result indicated that approximately 82% of
249 individuals neutralized one or more dengue serotypes by PRNT50. The proportion of
250 individuals with exposure to more than one serotype was found to be increased with age. They
251 were more likely to have been infected with DENV-1, followed by DENV-2, DENV-3, and
252 DENV-4.
253 The seroprevalence data among Thais individuals living in central and southern
254 Thailand (age ranged from 6 months to 60 years) indicated that the overall seropositive rate for
255 anti-DENV IgG antibodies by ELISA (catalog numbers EI266b-9601G, EUROIMMUN,
256 LuÈbeck, Germany) was 79%, which was similar to this study [10]. More recently, a higher
257 seroprevalence rate was observed in northeastern Thailand (approximately 90%, age ranged
258 from 5 to 93 years) by the same ELISA kit. Besides, the seroprevalence was 100% for
259 participants above the age of 25 years [11]. A previous study in healthy children in Bangkok
260 (the capital city of Thailand) indicated that 9-23% of children aged between 9-18 months had
261 neutralizing antibodies against one or more DENV serotypes, which were supposed to be
262 maternally transferred antibodies. DENV-1 was the most prevalent, followed by DENV-3,
263 DENV-2, and DENV-4 [14]. Accordingly, a recent study has investigated dengue antibodies
264 seroprevalence among mothers living in Ban Pong, Ratchaburi Province. The results showed
265 that 97% of mothers had dengue neutralizing antibodies to at least one dengue serotype in their
266 sera. Moreover, the proportion of cord sera with dengue neutralizing antibodies to all four
267 dengue serotypes was high at birth and similar to their mother's sera [15]. Based on these
268 results, the antibodies may have been of maternal origin. In older children, the antibodies might
269 be the result of exposure to infected mosquitoes.
270 As there is an increased risk of hospitalization [16] and severe dengue in seronegative
271 individuals, the WHO SAGE committee recommended using Dengvaxia only in scenes with
272 seroprevalence of approximately 70% or more at the age of 9 years [17]. Our data found that
273 the seropositive rate among 10-14 age groups was 68%. This indicated that such vaccination
274 programs might not be safe and effective in the Thai population. When tetravalent dengue
275 vaccines become available for use in dengue-endemic countries, it should be carefully
276 considered whether to target children who are nine years old or older than that.
277 This study also shows that both RDT and ELISA have very high specificity for
278 identifying PDI. The RDT demonstrated low sensitivity (35%), while the ELISA displayed
279 greater sensitivity (87%). Both RDTs and ELISA are widely used for the diagnosis of DENV
280 infection [18]. Dengue RDTs are convenient for surveillance in resource-limited settings since
281 they require fewer essential laboratory equipment. Currently, no study evaluated the
282 performance of RDT (SD Bioline) for the past DENV infection. A recent systematic review
283 indicated that the sensitivity and specificity of dengue IgG RDTs were typically above 75%
284 and 80%, respectively, compared with IgG ELISA testing in acute secondary DENV infections
285 and convalescent time points after recent illness [19].
286 Bonaparte M. et al. evaluated the performance of RDT (Biocan Diagnostics Inc) and
287 ELISA (SciMedx) in identifying individuals with PDI in Puerto Rico, using the PRNT50 as
288 the reference test. Both RDT and ELISA showed high specificity (>99%) but moderate
289 sensitivities (61 and 76%, respectively) [20]. However, in our evaluation, the RDT showed a
290 disappointing sensitivity compared to ELISA. Based on the present study, upon applying the
291 RDT to screen PDI, almost 65% of those supposed to be seropositive will be classified as a
292 seronegative group. Eventually, they will miss the opportunity for dengue vaccination. This
293 suggests that the dengue IgG RDT (SD Bioline) may not appropriate for identifying PDI,
294 especially in the endemic areas. However, the desirable characteristics of dengue pre-
295 vaccination screening tests have been debated [21, 22]. It has recently been recommended that
296 the desired sensitivity and specificity to be at least 90% [23]. Recently, there is a newly
297 developed, automated immunoglobulin fluorescence immunoassay for determining dengue
298 serostatus. However, its specificity and sensitivity are still insufficient for pre-vaccination
299 screening [24].
300 IgG ELISA measures total anti-DENV binding antibodies, whereas the PRNT measures
301 the neutralization capacity of the antibody response [6], which would be a part of binding
302 antibody and more specific to a particular virus than ELISA. The IgG ELISA might be more
303 sensitive and less specific as it also detects non-neutralizing and cross-reactive antibodies to
304 other flaviviruses within a serum specimen [25]. However, we found that those who were tested
305 positive for dengue IgG ELISA also were tested positive for PRNT, which would minimize the
306 likelihood of false positives in settings with past or current co-circulation with other
307 flaviviruses. Doum D. et al. reported that the IgG ELISA optical densities (OD) with age were
308 consistently higher in rural settings (R^2^=0.46-0.54) and lower in rural settings (R^2^=0.15-0.28)
309 [11]. Our urban study indicated that the IgG ELISA titers (RU/ml) with age were low
310 (R^2^=0.14). Moreover, we found a positive correlation between IgG ELISA titers and NT50
311 titers.
312 There are, however, several limitations in our study. First, the sample collection was
313 limited to the Ratchaburi province, which may not reflect the whole population. Second,
314 children under ten years old were not available in this study. Hence, we could not determine
315 the seroprevalence among 9-year-old children, which was also the targeted age for vaccination.
316 Third, we tested only one RDT (SD Bioline) and one ELISA kit (EUROIMMUN). It should
317 be noted that the sensitivity for identifying PDI may vary among different companies. Despite
318 these limitations, this study provides the most recent seroprevalence data for dengue viruses
319 from the western part of Thailand. We performed an age-stratified serosurvey using PRNT50,
320 distinguishing specific DENV serotypes and representing the true incidence of immunity to
321 DENV. Moreover, the RDT and ELISA performance in identifying PDI was demonstrated,
322 322
323 323

which might be used to determine their suitability for pre-vaccination screening.

# 324 Conclusions

325 In conclusion, this study confirms the distribution of multiple dengue serotypes in
326 Thailand. The frequency of DENV infection was increased in a time-dependent manner as the
327 high prevalence of multitypic DENV serotypes was observed in the older age group. Based on
328 the performance evaluation indicated that, if we use the RDT to screen PDI, almost 65% of
329 those supposed to be seropositive will miss the opportunity for dengue vaccination. Anti-
330 Dengue IgG ELISA is more suitable than the RTD for vaccine introduction and identifying
331 which populations to target.

# 332 Acknowledgments

333 We are grateful to the volunteer children and their parents for participating in the study.
334 We thank the medical staff at Banpong hospital and Potharam hospital for their support of this
335 project. We also thank Stefan Fernandez and the laboratory staff of the Department of
336 Virology, Armed Forces Research Institute of Medical Sciences (Bangkok, Thailand) for
337 337
338 338

PRNT assay training and reagents.

# 339 Reference:

340 1.	Lanciotti RS, Gubler DJ, Trent DW. Molecular evolution and phylogeny of dengue-4

341	viruses. J Gen Virol. 1997;78 (9):2279-84. Epub 1997/09/18. doi: 10.1099/0022-1317-78-9-

342	2279. PubMed PMID: 9292015.

343 2.	Rigau-Pérez JG, Clark GG, Gubler DJ, Reiter P, Sanders EJ, Vorndam AV. Dengue
344 and dengue haemorrhagic fever. Lancet. 1998;352(9132):971-7. Epub 1998/09/30. doi:

345	10.1016/s0140-6736(97)12483-7. PubMed PMID: 9752834.

346 3.	Sridhar S, Luedtke A, Langevin E, Zhu M, Bonaparte M, Machabert T, et al. Effect of
347 Dengue Serostatus on Dengue Vaccine Safety and Efficacy. N Engl J Med. 2018;379(4):327-

348	40. Epub 2018/06/14. doi: 10.1056/NEJMoa1800820. PubMed PMID: 29897841.

349	4.	World Health O. Dengue vaccine: WHO position paper, July 2016 - recommendations.

350	Vaccine. 2017;35(9):1200-1. Epub 2017/02/12. doi: 10.1016/j.vaccine.2016.10.070. PubMed

351	PMID: 28185744.

352 5.	Imrie A, Meeks J, Gurary A, Sukhbaatar M, Truong TT, Cropp CB, et al. Antibody to
353 dengue 1 detected more than 60 years after infection. Viral Immunol. 2007;20(4):672-5. Epub

354	2007/12/27. doi: 10.1089/vim.2007.0050. PubMed PMID: 18158740; PubMed Central

355 PMCID: PMCPMC3084288.
356 6.	Russell PK, Nisalak A, Sukhavachana P, Vivona S. A plaque reduction test for dengue
357 virus neutralizing antibodies. J Immunol. 1967;99(2):285-90. Epub 1967/08/01. PubMed

358	PMID: 6031202.

359 7.	Roehrig JT, Hombach J, Barrett AD. Guidelines for Plaque-Reduction Neutralization
360 Testing of Human Antibodies to Dengue Viruses. Viral Immunol. 2008;21(2):123-32. Epub

361	2008/05/15. doi: 10.1089/vim.2008.0007. PubMed PMID: 18476771.

362 8.	Peeling RW, Artsob H, Pelegrino JL, Buchy P, Cardosa MJ, Devi S, et al. Evaluation
363 of diagnostic tests: dengue. Nat Rev Microbiol. 2010;8(12 Suppl):S30-8. Epub 2011/05/07.

364	doi: 10.1038/nrmicro2459. PubMed PMID: 21548185.

365 9.	Malhotra RK, Indrayan A. A simple nomogram for sample size for estimating
366 sensitivity and specificity of medical tests. Indian J Ophthalmol. 2010;58(6):519-22. Epub

367	2010/10/19. doi: 10.4103/0301-4738.71699. PubMed PMID: 20952837; PubMed Central

368 PMCID: PMCPMC2993983.
369 10.	Vongpunsawad S, Intharasongkroh D, Thongmee T, Poovorawan Y. Seroprevalence of
370 antibodies to dengue and chikungunya viruses in Thailand. PLoS One. 2017;12(6):e0180560.

371	Epub 2017/07/01. doi: 10.1371/journal.pone.0180560. PubMed PMID: 28662144; PubMed

372 Central PMCID: PMCPMC5491253.
373 11.	Doum D, Overgaard HJ, Mayxay M, Suttiprapa S, Saichua P, Ekalaksananan T, et al.
374 Correction: Doum, D., et al. Dengue Seroprevalence and Seroconversion in Urban and Rural
375 Populations in Northeastern Thailand and Southern Laos. Int. J. Environ. Res. Public Health

376	2020, 17, 9134. Int J Environ Res Public Health. 2021;18(4). Epub 2021/02/10. doi:

377	10.3390/ijerph18041439.	PubMed	PMID:	33557444;	PubMed	Central	PMCID:

378	PMCPMC7913739.

379 12.	Endy TP, Nisalak A, Chunsuttitwat S, Vaughn DW, Green S, Ennis FA, et al.
380 Relationship of preexisting dengue virus (DV) neutralizing antibody levels to viremia and
381 severity of disease in a prospective cohort study of DV infection in Thailand. J Infect Dis.

382	2004;189(6):990-1000. Epub 2004/03/05. doi: 10.1086/382280. PubMed PMID: 14999601.

383 13.	Jang WS, Kwak SY, May WL, Yang DJ, Nam J, Lim CS. Comparative evaluation of
384 three dengue duo rapid test kits to detect NS1, IgM, and IgG associated with acute dengue in

385	children	in	Myanmar.	PLoS	One.	2019;14(3):e0213451.	Epub	2019/03/14.	doi:

386	10.1371/journal.pone.0213451. PubMed	PMID: 30865680; PubMed	Central	PMCID:

387	PMCPMC6415848.

388 14.	Pengsaa	K,	Limkittikul	K,	Luxemburger	C,	Yoksan	S,	Chambonneau	L,
389 Ariyasriwatana C, et al. Age-specific prevalence of dengue antibodies in Bangkok infants and
390 children.	Pediatr	Infect	Dis	J.	2008;27(5):461-3.	Epub	2008/03/25.	doi:

391	10.1097/INF.0b013e3181646d45. PubMed PMID: 18360303.

392 15.	Khamim K, Hattasingh W, Nisalak A, Kaewkungwal J, Fernandez S, Thaisomboonsuk
393 B, et al. Neutralizing dengue antibody in pregnant Thai women and cord blood. PLoS Negl

394	Trop Dis. 2015;9(2):e0003396. Epub 2015/02/07. doi: 10.1371/journal.pntd.0003396. PubMed

395 PMID: 25658481; PubMed Central PMCID: PMCPMC4320096.
396 16.	Hadinegoro SR, Arredondo-García JL, Capeding MR, Deseda C, Chotpitayasunondh
397 T, Dietze R, et al. Efficacy and Long-Term Safety of a Dengue Vaccine in Regions of Endemic

398	Disease.	N	Engl	J	Med.	2015;373(13):1195-206.	Epub	2015/07/28.	doi:

399	10.1056/NEJMoa1506223. PubMed PMID: 26214039.

400	17.	Dengue vaccine: WHO position paper, September 2018 - Recommendations. Vaccine.

401	2019;37(35):4848-9. Epub 2018/11/15. doi: 10.1016/j.vaccine.2018.09.063. PubMed PMID:

402	30424888.

403	18.	Raafat N, Blacksell SD, Maude RJ. A review of dengue diagnostics and implications

404	for surveillance and control. Trans R Soc Trop Med Hyg. 2019;113(11):653-60. Epub

405	2019/08/01. doi: 10.1093/trstmh/trz068. PubMed PMID: 31365115; PubMed Central PMCID:

406	PMCPMC6836713.

407	19.	Luo R, Fongwen N, Kelly-Cirino C, Harris E, Wilder-Smith A, Peeling RW. Rapid

408	diagnostic tests for determining dengue serostatus: a systematic review and key informant

409	interviews.	Clin	Microbiol	Infect.	2019;25(6):659-66.	Epub	2019/01/22.	doi:

| 410 | 10.1016/j.cmi.2019.01.002. | PubMed | PMID: | 30664935; PubMed | Central | PMCID: |
| --- | --- | --- | --- | --- | --- | --- |
| 411 | PMCPMC6543064. |  |  |  |  |  |

412	20.	Bonaparte M, Huleatt J, Hodge S, Zheng L, Lustig Y, DiazGranados CA, et al.

413	Evaluation of dengue serological tests available in Puerto Rico for identification of prior

414	dengue	infection	for	prevaccination	screening.	Diagn	Microbiol	Infect	Dis.

| 415 | 2020;96(3):114918. Epub 2019/12/17. doi: 10.1016/j.diagmicrobio.2019.114918. PubMed |
| --- | --- |
| 416 | PMID: 31839333. |
| 417 | 21. Rodríguez-Barraquer I, Salje H, Cummings DA. Dengue pre-vaccination screening and |
| 418 | positive predictive values. Lancet Infect Dis. 2019;19(2):132-4. Epub 2019/02/05. doi: |
| 419 | 10.1016/s1473-3099(18)30799-0. PubMed PMID: 30712834. |
| 420 | 22. Flasche S, Smith PG. Sensitivity and negative predictive value for a rapid dengue test. |
| 421 | Lancet Infect Dis. 2019;19(5):465-6. Epub 2019/04/30. doi: 10.1016/s1473-3099(19)30167-7. |
| 422 | PubMed PMID: 31034385. |
| 423 | 23. Wilder-Smith A, Smith PG, Luo R, Kelly-Cirino C, Curry D, Larson H, et al. Pre- |
| 424 | vaccination screening strategies for the use of the CYD-TDV dengue vaccine: A meeting |
| 425 | report. Vaccine. 2019;37(36):5137-46. Epub 2019/08/05. doi: 10.1016/j.vaccine.2019.07.016. |
| 426 | PubMed PMID: 31377079. |
| 427 | 24. Daag JV, Ylade M, Adams C, Jadi R, Crisostomo MV, Alpay R, et al. Evaluation of a |
| 428 | new point-of-care test to determine prior dengue infection for potential use in pre-vaccination |

429	screening. Clin Microbiol Infect. 2020. Epub 2020/09/01. doi: 10.1016/j.cmi.2020.08.026.

430	PubMed PMID: 32866651.

431	25.	WHO Guidelines Approved by the Guidelines Review Committee.	Dengue:

432	Guidelines for Diagnosis, Treatment, Prevention and Control: New Edition. Geneva: World

433

434

Health Organization. 2009.

STROBE checklist

[Click here to access/download](https://www.editorialmanager.com/pone/download.aspx?id=28656326&guid=40205465-0339-4de4-9c1c-3f9a86a99d2b&scheme=1)

[**Supporting Information** STROBEchecklist1.doc](https://www.editorialmanager.com/pone/download.aspx?id=28656326&guid=40205465-0339-4de4-9c1c-3f9a86a99d2b&scheme=1)
