# Supplementary material for: Dengue pre-vaccination screening test evaluation for the use of dengue vaccine in an endemic area
Source: PLoS One. 2021 Sep 10;16(9):e0257182. doi: 10.1371/journal.pone.0257182 (PMC8432984; doi:10.1371/journal.pone.0257182)
Supplement: Attachment — Submitted filename: Revised manuscripts_PLOS ONE-Final review.docx [file pone.0257182.s007.docx]

**Dengue pre-vaccination screening test evaluation for the use of dengue vaccine in an endemic area**

Umaporn Limothai^1,2^, Sasipha Tachaboon^1,2,^ Janejira Dinhuzen^1,2^, Taweewun Hunsawong^3^, Prapapun Ong-ajchaowlerd^3^, Butsaya Thaisomboonsuk^3^, Stefan Fernandez^3^, Supachoke Trongkamolchai^4^, Mananya Wanpaisitkul^4^, Chatchai Chulapornsiri^4^, Anongrat Tiawilai^5^, Thawat Tiawilai^5^, Terapong Tantawichien^6,7^, Usa Thisyakorn^7^, Nattachai Srisawat^1,2,7-10*^

1. Excellence Center for Critical Care Nephrology, King Chulalongkorn Memorial Hospital, Bangkok, Thailand.
2. Critical Care Nephrology Research Unit, Chulalongkorn University, Bangkok, Thailand.
3. Department of Virology, Armed Forces Research Institute of Medical Sciences, Bangkok, Thailand.
4. Banpong Hospital, Ratchaburi, Thailand.
5. Photharam Hospital, Ratchaburi, Thailand.
6. Division of Infectious Diseases, Department of Medicine, Faculty of Medicine, Chulalongkorn University, Bangkok, Thailand.
7. Tropical Medicine Cluster, Chulalongkorn University, Bangkok, Thailand.
8. Division of Nephrology, Department of Medicine, Faculty of Medicine, King Chulalongkorn Memorial Hospital, Bangkok 10330, Thailand.
9. Center for Critical Care Nephrology, The CRISMA Center, Department of Critical Care Medicine, University of Pittsburgh, School of Medicine, Pittsburgh, PA, USA.
10. Academy of Science, Royal Society of Thailand, Bangkok, Thailand.

**Keyword:** CYD-TDV; Dengvaxia; Dengue serostatus; Prior dengue infection

Prevaccination screening test; rapid diagnostic test; ELISA; Sensitivity; Specificity

**Short title:** Dengue pre-vaccination screening test evaluation

***Corresponding Author:** Assoc. Prof. Dr. Nattachai Srisawat

Email: [drnattachai@yahoo.com](mailto:drnattachai@yahoo.com)

# Abstract

**Background:** The dengue vaccine (Dengvaxia) is only recommended in individuals who have had prior dengue infection (PDI). In this study, we aimed to perform a serosurvey to support decision-making for vaccine introduction and identify which populations to target. We also evaluated the performance of the serological tests using plaque reduction neutralization test (PRNT) as a reference test in identifying PDI to determine their suitability for pre-vaccination screening.

**Methods:** We enrolled 115 healthy individuals between 10 and 22 years of age living in Ratchaburi province, Thailand. The serum samples were tested by PRNT to measure the prevalence and concentration of serotype-specific neutralizing antibodies. The performance of the IgG rapid diagnostic test (RDT, SD Bioline, Korea) and IgG enzyme-linked immunosorbent assay (ELISA, EUROIMMUN, Germany) in identifying PDI were evaluated by using PRNT as a reference method.

**Results:** Overall, 94 (81.7%) of individuals neutralized one or more dengue serotypes at a titer threshold of equal or more than 10. Multitypic profiles were observed in 70.4% of the samples, which increased to 91.9% in subjects aged 19-22. Amongst monotypic samples, the highest proportion was reactive against DENV-1, followed by DENV-2, DENV-3, and DENV-4. The highest anti-dengue antibody titers were recorded against DENV-1 and increased with age to a geometric mean NT50 titer (GMT) of 188.6 in the oldest age group. Both RDT and ELISA exhibited 100% specificity. However, the RDT demonstrated low sensitivity (35%), while the ELISA displayed greater sensitivity (87%).

**Conclusions:** Almost eighty percent of Thai adolescents and youth had already been exposed to the dengue virus. It is appropriate to mention that virus is circulating in Thailand. You analyzed the people in a provenance. All four dengue serotypes have been widely circulating in Thailand. The dengue IgG RDTs have shown low sensitivity and might not suitable for dengue pre-vaccination screening. Based on these results, IgG ELISA could safely guide vaccination in Thailand.

# Introduction

Dengue is a mosquito-borne viral disease that has rapidly spread across the tropical and subtropical regions. Dengue virus consists of four serotypes with antigenic differences (DENV-1 to 4) [1]. It has a broad clinical spectrum that includes both severe and non-severe clinical manifestations [2]. The first dengue vaccine, a live, attenuated, tetravalent dengue vaccine (Dengvaxia or CYD-TDV) developed by Sanofi Pasteur, was licensed and available in some countries. The efficacy trials (Please add some references here) showed that Dengvaxia protected against severe dengue in people who had exposure to dengue prior to vaccination. However, there was evidence of a higher longer-term risk of severe dengue in vaccinated persons who had not been exposed to dengue [3]. World Health Organization (WHO) Strategic Advisory Group of Experts (SAGE) recommends using Dengvaxia in children 9-16 years old living in highly dengue-endemic areas [4]. Furthermore, in defining people to be targeted for vaccination, prior dengue infection (PDI), as measured by seroprevalence, should be at least 80% by age 9 years [5]. To minimize potentially harmful effects, we should ensure that the age group targeted for vaccination has enough pre-existing dengue immunity to benefit from the vaccine [5].

Age-stratified serosurveys are currently the best method for selecting populations appropriate for vaccination. Immunoglobulin G (IgG)-class antibodies to dengue are useful serological markers for PDI as they can persist over an individual's lifetime [6]. Plaque reduction neutralization test (PRNT) is the most specific serological test for determining type-specific neutralizing antibodies to an infecting virus. This technique can be used to confirm the presence of the neutralization antibodies against Flaviviruses in the serum samples [7, 8]. However, PRNT is a labor-intensive, relatively costly test, and it might not be convenient for routine surveillance purposes (please add a reference). Commercial IgG rapid diagnostic test (RDT) and IgG enzyme-linked immunosorbent assay (ELISA) are commonly used to diagnose acute dengue infection [9]. Nonetheless, the performance data for these tests for identifying PDI are still limited [10]. A recent systematic review of RDTs showed sensitivities and specificities between 80% and 100% for dengue IgG detection compared with ELISA (please add the reference). However, these studies evaluated IgG detection in those with early convalescent samples or presumed acute primary or secondary infection, and no study evaluated the performance of RDTs for past DENV infection [10].

This study aimed to perform a comparative serosurvey to support decision-making for dengue vaccine introduction at nine years. We also evaluated the performance of the serological tests as a pre-screening test in identifying PDI to determine their suitability for pre-vaccination screening in Thailand.

# Materials and methods

## Ethics statement

The study was conducted according to the Helsinki Declaration and Good Clinical Practice guidelines. The study protocol was approved by the ethical committee of Banpong hospital (REC 008/2562) and Potharam hospital (REC 31/2562). The written informed consent was obtained from subjects ≥18 years old and parents of subjects <18 years old.

## Cells and Viruses

The PRNT assays were completed in LLC-MK2 (Rhesus monkey kidney cells) obtained from ATCC. The Cells were grown in M-199 supplement with 20% heated-inactivated fetal bovine serum (HIFBS, Invitrogen, US), 1× L-glutamine (Invitrogen, US), 100 units of penicillin-streptomycin (P&S, Invitrogen, US) at 35˚C with 5% CO_2_ incubator. A concentration of cells at 4×10^4^ per ml in M-199 with 10% HIFBS, 1× L-glutamine, 100 units of P&S was prepared and added 1.5 ml per well of 12-well plate, which continued cultured at 35˚C with 5% CO_2_ for 3 days before using in the PRNT assay. The four serotypes of DENV, including DENV-1 (16007), DENV-2 (16681), DENV-3 (16562), and DENV-4 (C0036/06), were used in the PRNT assay (Do you have the passage number of the viruses? It is important to add the passage number). Viruses were propagated in C6/36 mosquito cell line to obtain a sufficient titer (700PFU/ml) for testing. Virus titers were measure by plaque titration assay. Viruses were aliquot in small volume and stored at -70˚C until used without repeat freeze-thawing.You have to titer the virus after first freeze.

## Study Location and Population

The study was conducted in Banpong and Potharam district, Ratchaburi province, an urban center located in the western part of Thailand. Healthy children between 9 and 22 years of age at the enrollment and residing in Banpong or Potharam district were eligible to participate. Healthy children were defined as participants in good health based on medical history and physical examination. We excluded children with acute febrile illness (body temperature more than 37.5˚C) or moderate or severe acute illness/infection on the day of enrolment, according to the investigator's judgment. Using the sample size calculation by Malhotra RK. and Indrayan A. [11], assuming 91% seroprevalence [12, 13], expected sensitivity and specificity of 95% and 95%, respectively, and desired precision of 0.05, we estimate that at least 80 samples would need to be tested. With indeterminate and invalid results, we tested 115 samples. To examine the DENV seroprevalence in different age groups, we classified the participant's age into three categories based on school-age, namely 10-14 years, 15-18 years, and 19-22 years. Specimens were collected from August 2019-August 2020. The blood samples were collected in anticoagulant-free vacutainer tubes. The sera were separated and maintained refrigerated at 2-8˚C. The specimens were then transported on the day of collection to the central laboratory at Chulalongkorn hospital and stored at -80˚C before testing.

## Plaque reduction neutralization test (PRNT)

PRNT was performed to determine the levels of virus type-specific neutralizing (NT) antibodies using standard methods, as previously described [7, 14, 15]. Briefly, the serum was heat-inactivated at 56˚C for 30 minutes. In an ice bath, a 0.3 ml/tube of each 4-fold dilutions of the test sera beginning with a 1:10 dilution (1:10, 1:40, 1:160, 1:640 and 1:2,560) including a 0.3 ml serum diluent tube as a virus control well (MEM with 10%HIFBS) was mixed with an equal volume of each reference virus (DENV serotype noted above). Then, the virus-serum mixture was incubated in a 35°C water bath for 1 hour. The virus-serum mixture was then inoculated (0 .1 ml per well) into the 3-day old LLC-MK2 cells in duplicate wells of a 12-well plate and incubated at room temperature for 1 hour on a rocker platform. After removing of excess virus-serum mixture, the first overlay medium, containing 0.9% low-melting point agarose (LMP Ultra PureTM LMP agarose, Invitrogen, USA), in house Hank’s BSS, 1X Vitamins (Invitrogen, US), 1X amino acids (Invitrogen, US), 5%HIFBS, L-glutamine, and 7.5% sodium bicarbonate, was added (1 ml per well) and allowed to solidify for 15 minutes ast RT. Plates were incubated at 35°C, 5% CO_2_ incubator for 4-6 days. We observed the plates daily for the onset of CPEs? At the end of the incubation period, stained cells with a second overlay medium (0.9% LMP, in house Hank’s BSS, 1X Vitamins, and 1X amino acids) containing 4% Neutral red (Sigma, US) 1 ml per well, and allowed to solidify for 15 minutes. Plates were incubated overnight at 35°C in a 5% CO_2_ incubator before counting the number of plaques by manual counting. Positive control wells (virus without sera) were established for each assay run to ensure infectivity of the cell monolayer (formation of 25- 50 plaques/well was considered acceptable). Positive serum control was pooled high-titered convalescent sera against four dengue serotypes diluted with pooled normal human sera to get appropriate NT50 endpoint and aliquot in small volume and frozen storage for use. Plaque reduction neutralization endpoints were calculated using probit analysis. Briefly, the percentage of plaques counted in test sera was compared with the number of plaques from the control preparation. Log dilution of test sera preparation (i.e., 1:10, 1:40, 1:160, 1:640, 1:2,560) were plotted along the X-axis, whereas percent reduction in plaque count was detailed along the Y-axis. Percent count plaque reductions for each test sample at each dilution was plotted, and a best-fit line was drawn. The reciprocal of the lowest dilution of test sera to neutralize 50% of the control virus input represents the NT50 titer. Therefore, the NT50 titer is calculated by counting plaques and reporting the titer as the reciprocal of the last serum dilution to show a 50% reduction of the control plaque count based on the back-titration of control plaques. In this study, NT50 titers≥10 were considered positive for virus-neutralizing activity. An individual who has antibody titers <10 for the four serotypes was classified as naïve. An individual who has NT50 antibody titers ≥10 to only one serotype (no NT titer to other serotypes) was classified as monotypic. Correspondingly, an individual who has NT50 antibody titers ≥10 for more than one serotype was classified as multitypic.

## Dengue duo rapid test kit

Dengue nonstructural protein 1 (NS1) antigen and dengue IgG/IgM antibody were examined at the enrollment using SD Bioline Dengue Duo NS1 Ag & IgG/IgM (catalog number 11FK46, SD Bioline, Korea) according to the manufacturer's instructions. The kit contains two test devices; the left side is for the dengue NS1 antigen test, whereas the right side is for the dengue IgG/IgM test. The serum specimen was thawed to room temperature (15 °C - 30 °C) before use. For the NS1 antigen, 100 μL of undiluted serum sample was added to the left sample well, incubated at room temperature, and the results were read within 15-20 minutes. The presence of two colored lines including control line (C) and test line (T) in the result window indicates that the specimen is positive for the NS1 antigen whereas, the presence of only C line indicates that NS1 antigen was not present in the specimen or is present below the detectable levels. For IgG/IgM, 10 μL of undiluted serum sample was added to the right sample well. This was followed by adding four drops (90-120 μL) of assay diluent to the round assay diluent well and incubated at room temperature. The results were read 15-20 minutes later. The presence of two colored lines (C and M) in the result window indicates that the specimen is positive for IgM antibodies to dengue. The presence of two colored lines (C and G) indicates that the specimen is positive for IgG antibodies to dengue. In addition, the presence of three colored lines (C, M, and G) indicates that the specimen is positive for both IgM and IgG antibodies to dengue. In contrast, the presence of only the C line indicates that IgG and IgM antibodies to dengue are not present in the specimen or are present below the detectable levels [16]. The test results were investigated and interpreted by three different readers to avoid biases.

## Dengue IgG ELISA

Anti-Dengue IgG antibodies were assessed using commercial enzyme-linked immunosorbent assays (catalog number EI266b-9601G, EUROIMMUN, Lübeck, Germany) according to the manufacturer’s instructions. Briefly, serum samples were diluted at 1:101, and 100 μL of the mixture was then added to the 96-well microplates coated with purified DENV-2 particles. As the high structural similarity of DENV-1-4, using a single virus type is sufficient for the reliable detection of antibodies against all four virus types. After incubating for 60 minutes at 37°C, the wells were washed three times using 300 μl of working strength wash buffer for each wash, and 100 μL of enzyme conjugate (peroxidase-labeled anti-human IgG) was added to each well. After incubating for 30 minutes at room temperature, the wells were washed three times using 300 μl of working strength wash buffer for each wash, and the conjugated complex was visualized by adding 100 μL of chromogen/substrate solution into each of the microplate wells. After incubating for 15 minutes at room temperature (protect from direct sunlight), the reaction was stopped by adding 100 μl of stop solution into each microplate wells. Photometric measurement of the color intensity was made at a wavelength of 450 nm and a reference wavelength between 620 nm and 650 nm within 30 minutes of adding the stop solution. The IgG results were reported quantitatively according to the manufacturer’s instructions using the standard curve obtained by point-to-point plotting of the extinction values measured for the three calibration sera against the corresponding units (linear/linear). A specimen was considered IgG positive if the result was ≥22 relative units (RU)/ml, borderline if ≥16 and <22 RU/ml, and negative if <16 RU/ml.

## Statistical analysis

Descriptive statistics for seropositive rate and other characteristics were performed. Continuous variables are presented as the mean ± one standard deviation (SD) in case of a normal distribution and as a median and interquartile range (IQR) in case of non-normally distributed variables. The comparison was performed by one-way ANOVA or Kruskal–Wallis test as appropriate. Categorical variables were presented as numbers with percentages and were compared using the Chi-square test or Fisher’s exact test as appropriate. P-value <0.05 was considered significant. The geometric mean titer and the 95% CI for each age group and dengue serotype were calculated for all samples based on their DENV PRNT results. To calculate the GMT, samples with an NT50 titer <10 were given the value of 5. All analyses were performed using IBM SPSS Statistics software version 22.0 (IBM Corp., Armonk, NY) and figures were drawn using GraphPad Prism 8 (GraphPad Software Inc., California, USA).

## Disclaimer

Material has been reviewed by the Walter Reed Army Institute of Research. There is no objection to its presentation and/or publication. The opinions or assertions contained herein are the private views of the author, and are not to be construed as official, or as reflecting true views of the Department of the Army or the Department of Defense.

# Results

## Demographic data

Table 1 provides general characteristics of 115 participants included in the study. Their age ranged from 10 to 22 years, with a median of 16 years (IQR 13-20). More than half (67, 58.3%) were males. The participants were predominantly (53%) from Ban Pong District.

**Table 1 Demographic data**

| **Characteristics** | **Total (N=115)** |
| --- | --- |
| Gender, male, (N, %) | 67 (58.3) |
| Age, years (median, range) | 16 (10-22) |
| 10-14 (N,%) | 38 (33.0) |
| 15-18 (N,%) | 40 (34.8) |
| 19-22 (N,%) | 37 (32.2) |
| Area |  |
| Ban Pong (N,%) | 61 (53.0) |
| Potharam (N,%) | 54 (47.0) |

## Seroprevalence of DENV amongst participants

Serological evidence of previous exposure to DENV as denoted by NT50 titers ≥ 10 was identified in the serum samples of 94 (81.7%) participants. Seroprevalence did not significantly differ based on gender (*p*=0.113). To examine the DENV seroprevalence in different age groups, we classified the participant's age into three categories based on school-age, namely 10-14 years, 15-18 years, and 19-22 years. The data indicated that increasing age appeared to be associated with increased seropositivity (p=0.005). We also examined the seroprevalence by IgG ELISA and IgG rapid test. The results showed that the overall seropositive rate had decreased to 71.3% by IgG ELISA and 28.7% by IgG rapid test (Fig 1). WHO recommended the use of NT90 instead of NT50 in endemic areas to decrease the background serum cross-neutralization among Flaviviruses [17]. We also performed additional PRNT data analysis using a higher cut-off. The result showed that the overall seropositive decreased from 81.7% by NT50 to 68.7% by NT90 (S1 Table).

Based on the RDT, the result showed no positive NS1 result, indicating no early dengue infection case in this cohort. Moreover, there were 2 IgM positive cases, reflecting the asymptomatic acute primary dengue infection. There were 3 IgM and IgG positive cases, suggesting a late primary or early secondary dengue infection.

**Fig 1. Seroprevalence of DENV by PRNT, IgG ELISA, and IgG rapid test**

## Neutralizing antibody profile distribution stratified by age

Samples were categorized according to the NT50 profile. Multitypic profiles were observed in 70.4% of the subjects, with 55.3% in those aged 10-14 years old, 65.0% in the 15-18 years old, and 91.9% in the 19-22 years old (Fig 2).

**Fig 2. Prevalence of individuals with naïve (NT50 titer<10), monotypic (NT50 titer≥10 against to only one dengue serotype), or multitypic (NT50 titer≥10 against more than one serotypes) neutralizing antibody profile, by age group**

There were 31.6% naïve subjects in the 10-14 years old group, 20.0% of the 15-18 years old group and 2.7% of the 19-22 years old groups, and 18.3% of the overall sample had no detectable neutralizing dengue antibodies (NT50 titer<10, Fig 2).

Among monotypic samples, the highest proportion of samples were reactive against DENV-2, followed by DENV-1, DENV-3, and DENV-4, a trend observed in the 19-22 years old age groups. The proportion of samples that were reactive against DENV-1 and DENV-2 were evenly distributed amongst the 10–14 and 15–18 years old age groups.

The distribution of geometric mean NT50 titer (GMT) among the ages 10-22 years old was shown in Fig 3A. DENV-1 had the highest overall GMT (82.2), followed by DENV-2 (74.5), DENV-3 (59.3), and DENV-4 (34.2). The trends were also observed in the 10-14 years old age group (Fig 3B, the GMT were 43.1, 42.8, 38.8, and 25.8 respectively), and in the 19-22 years old age group (Fig 3D), the GMT was 188.6, 126.0, 124.9, and 51.7 respectively). For the 15-18 years old age groups (Fig 3C), DENV-2 had the highest GMT (77.7), followed by DENV-1 (70.5), DENV-3 (44.6), and DENV-4 (30.4).

**Fig 3. Dengue serotype-specific geometric mean NT50 titer (GMT) by age-group.**

The long solid line indicates the threshold for seropositive (an NT50 titer ≥10).

## Performance of the dengue IgG rapid test and dengue IgG ELISA for determination of dengue serostatus

We assessed the performance of the IgG rapid test and IgG ELISA for detection of dengue pre-exposure using the PRNT as the reference assay. Of the 94 patients classified as seropositive by NT50 (titers ≥ 10), 61 (64.9%) were tested negative by dengue IgG rapid test, and 12 (12.8%) were tested negative by dengue IgG ELISA. For the dengue IgG rapid test, sensitivity was 35.1%, and specificity was 100%. When we evaluated the dengue IgG ELISA to detect prior dengue infection, specificity was unchanged, but sensitivity increased to 87.2% compared to IgG rapid test. Positive predictive values (PPV) for both tests were equal (100%). Negative predictive values (NPV) were 25.6% and 63.6% for dengue rapid test and IgG ELISA, respectively (Table 2).

**Table 2. Performance of the dengue IgG rapid test and dengue IgG ELISA for dengue serostatus determination using the plaque reduction neutralization test (PRNT) as the reference assay.**

| **Test** | **Dengue rapid test (IgG)** | | **Dengue ELISA (IgG)** | |
| --- | --- | --- | --- | --- |
|  | Positive | Negative | Positive | Negative |
| Positive PRNT (n,%) | 33 (35.1%) | 61 (64.9%) | 82 (87.2%) | 12 (12.8%) |
| Negative PRNT (n,%) | 0 (0.0%) | 21 (100.0%) | 0 (0.0) | 21 (100.0%) |
| Sensitivity | 35.1% | | 87.2% | |
| Specificity | 100.0% | | 100.0% | |
| Positive predictive value | 100.0% | | 100.0% | |
| Negative predictive value | 25.6% | | 63.6% | |

The relationship between NT50 titer and dengue IgG level (relative RU/mL by ELISA) was also examined. The result showed that NT50 titer had a positive correlation with IgG level (r=0.701, p<0.001 for DENV-1, r=0.771, p<0.001 for DENV-2, r=0.667, p<0.001 for DENV-3, and r=0.725, p<0.001 for DENV-4). The distribution of dengue IgG level and NT50 titer by age was demonstrated in Fig 4.

**Fig 4. Distribution of dengue IgG level (relative units per milliliter by ELISA) and NT50 titer by age**

The dashed line indicates the threshold for dengue IgG ELISA (20 RU/ml) and NT50 (a titer of 10).

# Discussion

We conducted a dengue seroprevalence study, which identified serological evidence for the circulation of all four dengue serotypes among adolescents and youth in Ratchaburi province, Thailand. This province is located approximately 100 km west of Bangkok. It lies between the Maeklong River on the east and the Thai-Myanmar border on the west. We have a collaborative research network in this province. A previous dengue disease surveillance study on approximately 3000 primary-school children in seven schools in Muang district of Ratchaburi province indicated that approximately four percent of the children had laboratory-confirmed dengue per year, and all four dengue virus types were found to be the causes of illness in children in all seven schools. Moreover, the study pointed out that the Muang district of Ratchaburi province could be suitable for dengue vaccine testing [18]. Our study generated data on serotype-specific prevalence in areas where little data were previously available. The result indicated that approximately 82% of individuals neutralized one or more dengue serotypes shown by PRNT (NT50 titers ≥ 10). The proportion of individuals with exposure to more than one serotype was found to increase with age. The predominant serotypes were DEN-1 and DEN-2 in the 10–14 and 15–18 age groups born during 2003-2011. Similar to the previous report, DENV-1 and DENV-2 were the most commonly reported serotypes in Thailand's national and/or regional studies between 2000 and 2010 [19]. Besides, DEN-2 was predominant in the 19-22 years old age groups born during 1999-2002. This result was consistent with a previous surveillance study in the Ratchaburi province, which indicated that the predominant serotypes were DEN-2 in 1999 and 2002 [20].

The seroprevalence data among Thai individuals living in central and southern Thailand (age ranged from 6 months to 60 years) indicated that the overall seropositive rate for anti-DENV IgG antibodies by ELISA (catalog numbers EI266b-9601G, EUROIMMUN, Lübeck, Germany) was 79%, which was similar to this study [12]. More recently, a higher seroprevalence rate was observed in northeastern Thailand (approximately 90%, age ranged from 5 to 93 years) by the same ELISA kit. Besides, the seroprevalence was 100% for participants above the age of 25 years [21]. A previous study in healthy children in Bangkok (the capital city of Thailand) indicated that 9-23% of children aged between 9-18 months had neutralizing antibodies against one or more DENV serotypes, which were supposed to be maternally transferred antibodies. DENV-1 was the most prevalent, followed by DENV-3, DENV-2, and DENV-4 [22]. Accordingly, a recent study has investigated dengue antibodies seroprevalence among mothers living in Ban Pong, Ratchaburi Province. The results showed that 97% of mothers had dengue neutralizing antibodies to at least one dengue serotype in their sera. Moreover, the proportion of cord sera with dengue neutralizing antibodies to all four dengue serotypes was high at birth and similar to their mother's sera [23]. Based on these results, the antibodies may have been of maternal origin. In older children, the antibodies might be the result of exposure to infected mosquitoes.

As there is an increased risk of hospitalization [24] and severe dengue in seronegative individuals, the WHO SAGE committee recommended using Dengvaxia only in regions with seroprevalence of approximately 80% or more at the age of 9 years [5]. Our data found that the seropositive rate among 10-14 age groups was 68%. This indicated that such vaccination programs might not be safe and effective in the Thai population. When tetravalent dengue vaccines become available for use in dengue-endemic countries, it should be carefully considered whether to target children who are nine years old or older than that.

This study also shows that both RDT and ELISA have very high specificity in detecting IgG for identifying PDI. However, the RDT for detecting IgG demonstrated low sensitivity (35%), while the ELISA displayed greater sensitivity (87%). In addition, the PPV for both tests was equal (100%), indicating that the probability that a person with a positive test will be truly seropositive was 100%. Therefore, both RDT and ELISA could be used to ensure who will be safe from the adverse effects of the vaccination. In contrast, the NPV was 25% and 63% for the RDT and ELISA. The probability that an individual with a negative RDT test will be truly seronegative was only 25%, and the rest of 75% may be seropositive. Therefore, if we use the RDT to identify PDI, those with a negative RDT test result will have a 75% chance of losing the opportunity from the vaccination.

According to WHO recommendation [5], the screening tests should have a high specificity to avoid vaccinating truly seronegative persons. Moreover, the test should have a high sensitivity to ensure that a high proportion of seropositive persons are vaccinated.

Both RDTs and ELISA are widely used for the diagnosis of DENV infection [25]. Dengue RDTs are convenient for surveillance in resource-limited settings since they require fewer essential laboratory equipment. Currently, no study evaluated the performance of RDT (SD Bioline) for the past DENV infection. A recent systematic review indicated that the sensitivity and specificity of dengue IgG RDTs were typically above 75% and 80%, respectively, compared with IgG ELISA testing in acute secondary DENV infections and convalescent time points after recent illness [10].

Bonaparte M. et al. evaluated the performance of RDT (Biocan Diagnostics Inc) and ELISA (SciMedx) in identifying individuals with PDI in Puerto Rico, using the PRNT as the reference test. Both RDT and ELISA showed high specificity (>99%) but moderate sensitivities (61 and 76%, respectively) [26]. However, in our evaluation, the RDT showed a disappointing sensitivity compared to ELISA. Based on the present study, upon applying the RDT to screen PDI, almost 65% of those supposed to be seropositive will be classified as a seronegative group. Eventually, they will miss the opportunity for dengue vaccination. This suggests that the dengue IgG RDT (SD Bioline) may not appropriate for identifying PDI, especially in the endemic areas. However, the desirable characteristics of dengue pre-vaccination screening tests have been debated [27, 28]. It has recently been recommended that the desired sensitivity and specificity to be at least 90% [29]. Recently, there is a newly developed, automated immunoglobulin fluorescence immunoassay for determining dengue serostatus. However, its specificity and sensitivity are still insufficient for pre-vaccination screening [30].

IgG ELISA measures total anti-DENV binding antibodies, whereas the PRNT measures the neutralization capacity of the antibody response [7], which would be a part of binding antibody and more specific to a particular virus than ELISA. The IgG ELISA might be more sensitive and less specific as it also detects non-neutralizing and cross-reactive antibodies to other flaviviruses within a serum specimen [31]. However, we found that those who were tested positive for dengue IgG ELISA also were tested positive for PRNT, which would minimize the likelihood of false positives in settings with past or current co-circulation with other flaviviruses. Doum D. et al. reported that the IgG ELISA optical densities (OD) with age were consistently higher in rural settings (R^2^=0.46-0.54) and lower in urban settings (R^2^=0.15-0.28) [13]. Our urban study indicated that the IgG ELISA titers (RU/ml) were low (R^2^=0.14). Moreover, we found a positive correlation between IgG ELISA titers and NT50 titers.

There are, however, several limitations in our study. First, the sample collection was limited to the Ratchaburi province, which may not reflect the whole population. Second, children under ten years old were not available in this study. Hence, we could not determine the seroprevalence among 9-year-old children, which was also the targeted age for vaccination. Third, we tested only one RDT (SD Bioline) and one ELISA kit (EUROIMMUN). It should be noted that the sensitivity for identifying PDI may vary among different companies. Third, it is important to realize that cross-reactivity of anti-DENV antibodies with other flaviviruses could not be excluded by most commercial antibody-based assays. Dengue and Japanese encephalitis virus (JEV) are in the same flavivirus family which shared common antigenic epitopes and high cross-reactivities are not uncommon. In Thailand, a mouse brain-derived-inactivated JEV vaccine was initially included in the routine immunization schedule in 1990. Then, it became a nationwide vaccination in 2000 in Thai children at 12–18 months of age [32]. For this reason, all participants in this study probably were vaccinated against JEV for at least nine years. However, the detailed vaccination history of all participants was not collected. Moreover, cases of JEV encephalitis have been reported even in vaccinated cases [33], so natural JEV infection might not be ruled out. Convalescent anti-dengue antibody responses after dengue infection in JEV vaccinated and individuals who lived in JEV endemic areas were dengue-specific and higher than anti-JEV as determined by PRNT [34]. A previous study showed that the sera of patients with JEV showed cross-reactivity to DENV IgM and/or IgG ELISA, but the cross-reactivity was not detected in the neutralization tests against DENV [35]. In addition, Saito Y. et al. reported cross-reactivity to DENV in an anti-DENV IgG ELISA after JEV vaccination. However, the DENV neutralizing antibodies were not detected in post-JEV immunization samples [36], indicating that neutralization tests are more specific than ELISA. Therefore, the serological data obtained from this study should be interpreted with caution since the samples were obtained in Thailand, where several known flaviviruses have been reported.

Despite these limitations, this study provides the most recent seroprevalence data for dengue viruses from the western part of Thailand. We performed an age-stratified serosurvey using PRNT, distinguishing specific DENV serotypes and representing the true incidence of immunity to DENV. Moreover, the RDT and ELISA performance in identifying PDI was demonstrated, which might be used to determine their suitability for pre-vaccination screening.

# Conclusions

In conclusion, this study confirms the distribution of multiple dengue serotypes in Thailand. The frequency of DENV infection was increased in a time-dependent manner as the high prevalence of multitypic DENV serotypes was observed in the older age group. Due to their lower sensitivity of the RDT for identifying PDI, those with a negative RDT test result might lose the benefits of vaccination. Therefore, we do not recommend using the RDT to identify PDI in this specific setting. Anti-Dengue IgG ELISA is more suitable than the RTD for vaccine introduction and identifying which populations to target.

# Acknowledgments

We are grateful to the volunteer children and their parents for participating in the study. We thank the medical staff at Banpong hospital and Potharam hospital for their support of this project. We also thank Stefan Fernandez and the laboratory staff of the Department of Virology, Armed Forces Research Institute of Medical Sciences (Bangkok, Thailand) for PRNT assay training and reagents.

# Reference:

1.	Lanciotti RS, Gubler DJ, Trent DW. Molecular evolution and phylogeny of dengue-4 viruses. J Gen Virol. 1997;78 ( Pt 9):2279-84. Epub 1997/09/18. doi: 10.1099/0022-1317-78-9-2279. PubMed PMID: 9292015.

2.	Rigau-Pérez JG, Clark GG, Gubler DJ, Reiter P, Sanders EJ, Vorndam AV. Dengue and dengue haemorrhagic fever. Lancet. 1998;352(9132):971-7. Epub 1998/09/30. doi: 10.1016/s0140-6736(97)12483-7. PubMed PMID: 9752834.

3.	Sridhar S, Luedtke A, Langevin E, Zhu M, Bonaparte M, Machabert T, et al. Effect of Dengue Serostatus on Dengue Vaccine Safety and Efficacy. N Engl J Med. 2018;379(4):327-40. Epub 2018/06/14. doi: 10.1056/NEJMoa1800820. PubMed PMID: 29897841.

4.	World Health O. Dengue vaccine: WHO position paper, July 2016 - recommendations. Vaccine. 2017;35(9):1200-1. Epub 2017/02/12. doi: 10.1016/j.vaccine.2016.10.070. PubMed PMID: 28185744.

5.	Dengue vaccine: WHO position paper, September 2018 - Recommendations. Vaccine. 2019;37(35):4848-9. Epub 2018/11/15. doi: 10.1016/j.vaccine.2018.09.063. PubMed PMID: 30424888.

6.	Imrie A, Meeks J, Gurary A, Sukhbaatar M, Truong TT, Cropp CB, et al. Antibody to dengue 1 detected more than 60 years after infection. Viral Immunol. 2007;20(4):672-5. Epub 2007/12/27. doi: 10.1089/vim.2007.0050. PubMed PMID: 18158740; PubMed Central PMCID: PMCPMC3084288.

7.	Russell PK, Nisalak A, Sukhavachana P, Vivona S. A plaque reduction test for dengue virus neutralizing antibodies. J Immunol. 1967;99(2):285-90. Epub 1967/08/01. PubMed PMID: 6031202.

8.	Roehrig JT, Hombach J, Barrett AD. Guidelines for Plaque-Reduction Neutralization Testing of Human Antibodies to Dengue Viruses. Viral Immunol. 2008;21(2):123-32. Epub 2008/05/15. doi: 10.1089/vim.2008.0007. PubMed PMID: 18476771.

9.	Peeling RW, Artsob H, Pelegrino JL, Buchy P, Cardosa MJ, Devi S, et al. Evaluation of diagnostic tests: dengue. Nat Rev Microbiol. 2010;8(12 Suppl):S30-8. Epub 2011/05/07. doi: 10.1038/nrmicro2459. PubMed PMID: 21548185.

10.	Luo R, Fongwen N, Kelly-Cirino C, Harris E, Wilder-Smith A, Peeling RW. Rapid diagnostic tests for determining dengue serostatus: a systematic review and key informant interviews. Clin Microbiol Infect. 2019;25(6):659-66. Epub 2019/01/22. doi: 10.1016/j.cmi.2019.01.002. PubMed PMID: 30664935; PubMed Central PMCID: PMCPMC6543064.

11.	Malhotra RK, Indrayan A. A simple nomogram for sample size for estimating sensitivity and specificity of medical tests. Indian J Ophthalmol. 2010;58(6):519-22. Epub 2010/10/19. doi: 10.4103/0301-4738.71699. PubMed PMID: 20952837; PubMed Central PMCID: PMCPMC2993983.

12.	Vongpunsawad S, Intharasongkroh D, Thongmee T, Poovorawan Y. Seroprevalence of antibodies to dengue and chikungunya viruses in Thailand. PLoS One. 2017;12(6):e0180560. Epub 2017/07/01. doi: 10.1371/journal.pone.0180560. PubMed PMID: 28662144; PubMed Central PMCID: PMCPMC5491253.

13.	Doum D, Overgaard HJ, Mayxay M, Suttiprapa S, Saichua P, Ekalaksananan T, et al. Dengue Seroprevalence and Seroconversion in Urban and Rural Populations in Northeastern Thailand and Southern Laos. Int J Environ Res Public Health. 2020;17(23). Epub 2020/12/11. doi: 10.3390/ijerph17239134. PubMed PMID: 33297445; PubMed Central PMCID: PMCPMC7731008.

14.	Endy TP, Nisalak A, Chunsuttitwat S, Vaughn DW, Green S, Ennis FA, et al. Relationship of preexisting dengue virus (DV) neutralizing antibody levels to viremia and severity of disease in a prospective cohort study of DV infection in Thailand. J Infect Dis. 2004;189(6):990-1000. Epub 2004/03/05. doi: 10.1086/382280. PubMed PMID: 14999601.

15.	Thomas SJ, Nisalak A, Anderson KB, Libraty DH, Kalayanarooj S, Vaughn DW, et al. Dengue plaque reduction neutralization test (PRNT) in primary and secondary dengue virus infections: How alterations in assay conditions impact performance. Am J Trop Med Hyg. 2009;81(5):825-33. Epub 2009/10/29. doi: 10.4269/ajtmh.2009.08-0625. PubMed PMID: 19861618; PubMed Central PMCID: PMCPMC2835862.

16.	Jang WS, Kwak SY, May WL, Yang DJ, Nam J, Lim CS. Comparative evaluation of three dengue duo rapid test kits to detect NS1, IgM, and IgG associated with acute dengue in children in Myanmar. PLoS One. 2019;14(3):e0213451. Epub 2019/03/14. doi: 10.1371/journal.pone.0213451. PubMed PMID: 30865680; PubMed Central PMCID: PMCPMC6415848.

17.	World Health O. Guidelines for plaque reduction neutralization testing of human antibodies to dengue viruses. Geneva: World Health Organization; 2007.

18.	Sabchareon A, Sirivichayakul C, Limkittikul K, Chanthavanich P, Suvannadabba S, Jiwariyavej V, et al. Dengue infection in children in Ratchaburi, Thailand: a cohort study. I. Epidemiology of symptomatic acute dengue infection in children, 2006-2009. PLoS Negl Trop Dis. 2012;6(7):e1732. Epub 2012/08/04. doi: 10.1371/journal.pntd.0001732. PubMed PMID: 22860141; PubMed Central PMCID: PMCPMC3409110.

19.	Limkittikul K, Brett J, L'Azou M. Epidemiological trends of dengue disease in Thailand (2000-2011): a systematic literature review. PLoS Negl Trop Dis. 2014;8(11):e3241. Epub 2014/11/07. doi: 10.1371/journal.pntd.0003241. PubMed PMID: 25375766; PubMed Central PMCID: PMCPMC4222696.

20.	Anantapreecha S, Chanama S, A-nuegoonpipat A, Naemkhunthot S, Sa-ngasang A, Sawanpanyalert P, et al. Annual Changes of Predominant Dengue Virus Serotypes in Six Regional Hospitals in Thailand from 1999 to 2002. Dengue Bulletin. 2004;28:1–6.

21.	Doum D, Overgaard HJ, Mayxay M, Suttiprapa S, Saichua P, Ekalaksananan T, et al. Correction: Doum, D., et al. Dengue Seroprevalence and Seroconversion in Urban and Rural Populations in Northeastern Thailand and Southern Laos. Int J Environ Res Public Health. 2021;18(4). Epub 2021/02/10. doi: 10.3390/ijerph18041439. PubMed PMID: 33557444; PubMed Central PMCID: PMCPMC7913739.

22.	Pengsaa K, Limkittikul K, Luxemburger C, Yoksan S, Chambonneau L, Ariyasriwatana C, et al. Age-specific prevalence of dengue antibodies in Bangkok infants and children. Pediatr Infect Dis J. 2008;27(5):461-3. Epub 2008/03/25. doi: 10.1097/INF.0b013e3181646d45. PubMed PMID: 18360303.

23.	Khamim K, Hattasingh W, Nisalak A, Kaewkungwal J, Fernandez S, Thaisomboonsuk B, et al. Neutralizing dengue antibody in pregnant Thai women and cord blood. PLoS Negl Trop Dis. 2015;9(2):e0003396. Epub 2015/02/07. doi: 10.1371/journal.pntd.0003396. PubMed PMID: 25658481; PubMed Central PMCID: PMCPMC4320096.

24.	Hadinegoro SR, Arredondo-García JL, Capeding MR, Deseda C, Chotpitayasunondh T, Dietze R, et al. Efficacy and Long-Term Safety of a Dengue Vaccine in Regions of Endemic Disease. N Engl J Med. 2015;373(13):1195-206. Epub 2015/07/28. doi: 10.1056/NEJMoa1506223. PubMed PMID: 26214039.

25.	Raafat N, Blacksell SD, Maude RJ. A review of dengue diagnostics and implications for surveillance and control. Trans R Soc Trop Med Hyg. 2019;113(11):653-60. Epub 2019/08/01. doi: 10.1093/trstmh/trz068. PubMed PMID: 31365115; PubMed Central PMCID: PMCPMC6836713.

26.	Bonaparte M, Huleatt J, Hodge S, Zheng L, Lustig Y, DiazGranados CA, et al. Evaluation of dengue serological tests available in Puerto Rico for identification of prior dengue infection for prevaccination screening. Diagn Microbiol Infect Dis. 2020;96(3):114918. Epub 2019/12/17. doi: 10.1016/j.diagmicrobio.2019.114918. PubMed PMID: 31839333.

27.	Rodríguez-Barraquer I, Salje H, Cummings DA. Dengue pre-vaccination screening and positive predictive values. Lancet Infect Dis. 2019;19(2):132-4. Epub 2019/02/05. doi: 10.1016/s1473-3099(18)30799-0. PubMed PMID: 30712834.

28.	Flasche S, Smith PG. Sensitivity and negative predictive value for a rapid dengue test. Lancet Infect Dis. 2019;19(5):465-6. Epub 2019/04/30. doi: 10.1016/s1473-3099(19)30167-7. PubMed PMID: 31034385.

29.	Wilder-Smith A, Smith PG, Luo R, Kelly-Cirino C, Curry D, Larson H, et al. Pre-vaccination screening strategies for the use of the CYD-TDV dengue vaccine: A meeting report. Vaccine. 2019;37(36):5137-46. Epub 2019/08/05. doi: 10.1016/j.vaccine.2019.07.016. PubMed PMID: 31377079.

30.	Daag JV, Ylade M, Adams C, Jadi R, Crisostomo MV, Alpay R, et al. Evaluation of a new point-of-care test to determine prior dengue infection for potential use in pre-vaccination screening. Clin Microbiol Infect. 2020. Epub 2020/09/01. doi: 10.1016/j.cmi.2020.08.026. PubMed PMID: 32866651.

31.	WHO Guidelines Approved by the Guidelines Review Committee. Dengue: Guidelines for Diagnosis, Treatment, Prevention and Control: New Edition. Geneva, World Health Organization. 2009.

32.	Tsai TF. New initiatives for the control of Japanese encephalitis by vaccination: minutes of a WHO/CVI meeting, Bangkok, Thailand, 13-15 October 1998. Vaccine. 2000;18 Suppl 2:1-25. Epub 2000/05/24. doi: 10.1016/s0264-410x(00)00037-2. PubMed PMID: 10821969.

33.	Olsen SJ, Supawat K, Campbell AP, Anantapreecha S, Liamsuwan S, Tunlayadechanont S, et al. Japanese encephalitis virus remains an important cause of encephalitis in Thailand. Int J Infect Dis. 2010;14(10):e888-92. Epub 2010/08/03. doi: 10.1016/j.ijid.2010.03.022. PubMed PMID: 20674433.

34.	Yamada K, Takasaki T, Nawa M, Yabe S, Kurane I. Antibody responses determined for Japanese dengue fever patients by neutralization and hemagglutination inhibition assays demonstrate cross-reactivity between dengue and Japanese encephalitis viruses. Clin Diagn Lab Immunol. 2003;10(4):725-8. Epub 2003/07/11. doi: 10.1128/cdli.10.4.725-728.2003. PubMed PMID: 12853413; PubMed Central PMCID: PMCPMC164242.

35.	Maeki T, Tajima S, Ikeda M, Kato F, Taniguchi S, Nakayama E, et al. Analysis of cross-reactivity between flaviviruses with sera of patients with Japanese encephalitis showed the importance of neutralization tests for the diagnosis of Japanese encephalitis. J Infect Chemother. 2019;25(10):786-90. Epub 2019/05/21. doi: 10.1016/j.jiac.2019.04.003. PubMed PMID: 31105002.

36.	Saito Y, Moi ML, Takeshita N, Lim CK, Shiba H, Hosono K, et al. Japanese encephalitis vaccine-facilitated dengue virus infection-enhancement antibody in adults. BMC Infect Dis. 2016;16(1):578. Epub 2016/10/21. doi: 10.1186/s12879-016-1873-8. PubMed PMID: 27756212; PubMed Central PMCID: PMCPMC5070094.

**Supporting information**

**S1 Table. Seroprevalence of DENV by PRNT (NT50, NT80 and NT90), IgG ELISA, and IgG rapid test.**
