# Supplementary material for: Dengue pre-vaccination screening test evaluation for the use of dengue vaccine in an endemic area
Source: PLoS One. 2021 Sep 10;16(9):e0257182. doi: 10.1371/journal.pone.0257182 (PMC8432984; doi:10.1371/journal.pone.0257182)
Supplement: Attachment — Submitted filename: Response to reviewer_R2.docx [file pone.0257182.s008.docx]

**PONE-D-21-09490**

**Dengue pre-vaccination screening test evaluation for the use of dengue vaccine in an endemic area**

**Journal Requirements:** Please review your reference list to ensure that it is complete and correct. If you have cited papers that have been retracted, please include the rationale for doing so in the manuscript text, or remove these references and replace them with relevant current references. Any changes to the reference list should be mentioned in the rebuttal letter that accompanies your revised manuscript. If you need to cite a retracted article, indicate the article’s retracted status in the References list and also include a citation and full reference for the retraction notice.

**Response** We have reviewed and revised references as suggested. We confirmed that no retracted article was cited in this manuscript. We removed the references “Doum D, Overgaard HJ, Mayxay M, Suttiprapa S, Saichua P, Ekalaksananan T, et al. Correction: Doum, D., et al. Dengue Seroprevalence and Seroconversion in Urban and Rural Populations in Northeastern Thailand and Southern Laos. Int J Environ Res Public Health. 2021;18(4).” and replaced them with “Doum D, Overgaard HJ, Mayxay M, Suttiprapa S, Saichua P, Ekalaksananan T, et al. Dengue Seroprevalence and Seroconversion in Urban and Rural Populations in Northeastern Thailand and Southern Laos. Int J Environ Res Public Health. 2020;17(23).” Moreover, following references were added in this revision as the reviewer's suggestion.

- Ref 3.	Hadinegoro SR, Arredondo-García JL, Capeding MR, Deseda C, Chotpitayasunondh T, Dietze R, et al. Efficacy and Long-Term Safety of a Dengue Vaccine in Regions of Endemic Disease. N Engl J Med. 2015;373(13):1195-206.
- Ref 4.	Wilder-Smith A, Vannice KS, Hombach J, Farrar J, Nolan T. Population Perspectives and World Health Organization Recommendations for CYD-TDV Dengue Vaccine. J Infect Dis. 2016;214(12):1796-9.
- Ref 5.	Sridhar S, Luedtke A, Langevin E, Zhu M, Bonaparte M, Machabert T, et al. Effect of Dengue Serostatus on Dengue Vaccine Safety and Efficacy. N Engl J Med. 2018;379(4):327-40.
- Ref 10.	Roehrig JT, Hombach J, Barrett AD. Guidelines for Plaque-Reduction Neutralization Testing of Human Antibodies to Dengue Viruses. Viral Immunol. 2008;21(2):123-32.
- Ref 11.	Raafat N, Blacksell SD, Maude RJ. A review of dengue diagnostics and implications for surveillance and control. Trans R Soc Trop Med Hyg. 2019;113(11):653-60.
- Ref 13.	Luo R, Fongwen N, Kelly-Cirino C, Harris E, Wilder-Smith A, Peeling RW. Rapid diagnostic tests for determining dengue serostatus: a systematic review and key informant interviews. Clin Microbiol Infect. 2019;25(6):659-66.
- Ref 26.	Wilder-Smith A, Smith PG, Luo R, Kelly-Cirino C, Curry D, Larson H, et al. Pre-vaccination screening strategies for the use of the CYD-TDV dengue vaccine: A meeting report. Vaccine. 2019;37(36):5137-46.

**Reviewer #2:** I added some minor recommendations in the manuscript.

1. It is appropriate to mention that virus is circulating in Thailand. You analyzed the people in a provenance.

**Response** Thank you for the suggestion. We have revised the conclusion part as the following sentence.

“Almost 80% of adolescents and youth in Ratchaburi province had already been exposed to one or more of the dengue virus serotypes. The dengue IgG RDT displayed low sensitivity and is likely not be suitable for dengue pre-vaccination screening. These results support the use of IgG ELISA test for dengue vaccination in endemic areas.”

2. Please add some references in the introduction

**Response** Thank you for the comment. We have added references in the introduction section, page 4-5 as suggested.

“The efficacy trials showed that Dengvaxia protected against severe dengue in people who had exposure to dengue prior to vaccination.[3-5]”

“However, PRNT is a labor-intensive, relatively costly test making it inconvenient for routine surveillance purposes.[10, 11]”

“A recent systematic review of RDT showed sensitivities and specificities between 80% and 100% for dengue IgG detection compared to ELISA.[13]”

- Ref 3.	Hadinegoro SR, Arredondo-García JL, Capeding MR, Deseda C, Chotpitayasunondh T, Dietze R, et al. Efficacy and Long-Term Safety of a Dengue Vaccine in Regions of Endemic Disease. N Engl J Med. 2015;373(13):1195-206.
- Ref 4.	Wilder-Smith A, Vannice KS, Hombach J, Farrar J, Nolan T. Population Perspectives and World Health Organization Recommendations for CYD-TDV Dengue Vaccine. J Infect Dis. 2016;214(12):1796-9.
- Ref 5.	Sridhar S, Luedtke A, Langevin E, Zhu M, Bonaparte M, Machabert T, et al. Effect of Dengue Serostatus on Dengue Vaccine Safety and Efficacy. N Engl J Med. 2018;379(4):327-40.
- Ref 10.	Roehrig JT, Hombach J, Barrett AD. Guidelines for Plaque-Reduction Neutralization Testing of Human Antibodies to Dengue Viruses. Viral Immunol. 2008;21(2):123-32.
- Ref 11.	Raafat N, Blacksell SD, Maude RJ. A review of dengue diagnostics and implications for surveillance and control. Trans R Soc Trop Med Hyg. 2019;113(11):653-60.
- Ref 13.	Luo R, Fongwen N, Kelly-Cirino C, Harris E, Wilder-Smith A, Peeling RW. Rapid diagnostic tests for determining dengue serostatus: a systematic review and key informant interviews. Clin Microbiol Infect. 2019;25(6):659-66.

3. Do you have the passage number of the viruses? It is important to add the passage number

**Response** We have added the passage number of the viruses in the Materials and methods section, Cells and Viruses part, page 5-6 as suggested.

“The four serotypes of DENV including DENV-1 (16007-Passage SM-2 C6/36-5), DENV-2 (16681-Passage MIK2-3, C6/36-1, SM-2, C6/3), DENV-3 (16562-Passage MK2-3, C6/36-2, SM-1, C6/36-6) and DENV-4 (C0036/06-Passage C6/36-8) were used in the PRNT assay.”

4. You have to titer the virus after first freeze.

**Response** We have added this point in the Materials and methods section, Cells and Viruses part, page 6 as suggested.

“We titered the virus after the first freeze using a procedure similar to PRNT assay to determine virus titer and also for virus dilution to reach 70 PFU/100 ul.”

5. We observed the plates daily for the onset of CPEs?

**Response** CPEs observation has been done daily only during the step of virus propagation to ensure that we harvested virus at the right time. However, for PRNT assay we don’t do that as the appropriate incubation period has already been set up for a particular DENV serotype to get the suitable plaque size for counting.

6. Please send the manuscript for English editing.

**Response** The grammar was rechecked and corrected by the medical manuscript editor from Research Affairs, Faculty of Medicine Chulalongkorn University (Michael D. Ullman, Ph.D.).

7. You can make the discussion more better.

**Response** We very much appreciate reviewer's suggestion and we have revised the Discussion section by adding some sentences (See page 17 and 19) as follows.

“According to WHO recommendations [7], the screening tests should have a high specificity to minimize individual risk and the inadvertent use of the vaccine in seronegative persons who have false-positive test results. The test should also have high sensitivity to maximize individual and population benefit by identifying a high proportion of previously exposed persons who can benefit from vaccination. The Global Dengue and Aedes-transmitted diseases Consortium (GDAC) suggested that any decision about implementing a prevaccination screening strategy with the commercially available tests at the country level requires cautious evaluation. In a high seroprevalence area, a test with very high sensitivity is needed. In a low seroprevalence area, a very high specificity test is crucial; however, a national implementation may not be cost-effective.[26]”

“In this study, we used the ELISA index cutoff values for seropositivity as stated in the product insert. However, it was unclear whether the ELISA cutoff values recommended by the manufacturer were suitable for prevaccination screening.”
